# Pan-cancer analysis identified CD248 as a potential target for multiple tumor types

**DOI:** 10.3389/fphar.2025.1554632

**Published:** 2025-04-10

**Authors:** Li Guo, Yan Liao, Xuyang Zhang, Rongjuan Guo, Zheng Wang, Deqin Yang

**Affiliations:** ^1^ Chongqing Key Laboratory of Oral Diseases, Stomatological Hospital of Chongqing Medical University, Chongqing, China; ^2^ Department of Conservative Dentistry and Endodontics, Shanghai Stomatological Hospital and School of Stomatology, Fudan University, Shanghai, China; ^3^ Shanghai Key Laboratory of Craniomaxillofacial Development and Diseases, Fudan University, Shanghai, China

**Keywords:** CD248, pan-cancer, immune cell, overall survival, single-cell sequencing, targeted therapy

## Abstract

**Objective:**

Tumors remain a major cause of death worldwide due to late-stage presentation and late diagnosis. Cell therapies have revolutionized the landscape in the precision treatment of tumors. However, there are still many challenges that limit the therapeutic efficacy. Additionally, cancer treatment also entails a major financial burden throughout the entire phase, making it preferable to find a specific biomarker for the early prognosis of the tumor.

**Methods:**

In this study, the role of CD248 in pan-cancer was analyzed through diverse tumor-associated databases, such as the Human Protein Atlas Database, the GEPIA2 Database, the cBioPortal Database, the TIMER Database, the STRING tool, and so on. In addition, CD248 mRNA and protein levels were assessed in a series of head and neck squamous cell carcinoma (HNSC) cell lines using qRT-PCR and Western blot. Furthermore, siCD248 was used to detect the effect of CD248 on the invasion, migration, and proliferation of HNSC cells by transwell assay, scratch wound healing assay, and EdU assay, respectively.

**Results:**

CD248 expression was significantly increased and correlated with advanced stage and poor prognosis in various tumors. Genetic alterations of CD248 were also associated with a poor prognosis of patients. Single-cell sequencing revealed that CD248 was mainly expressed on fibroblasts within the stroma, and its expression was positively correlated with the infiltration of immune cells in tumors. In addition, CD248 interacted with 11 common tumor biomarkers. Experiment results indicated that CD248 mRNA and protein expression were upregulated in HNSC cell lines, and inhibition of CD248 suppresses the invasion, migration, and proliferation of HNSC cells.

**Conclusion:**

High CD248 expression played a crucial role in pan-cancer, including immune cell infiltration, tumor progression and metastasis, and patient prognosis. CD248 plays a crucial role in tumor cells’ functions, including invasion, migration, and proliferation. All these findings indicated that CD248 may be a novel oncoprotein and a potential therapeutic target for pan-cancer.

## 1 Introduction

Tumors still pose a significant threat to human health due to their rapid growth and progression. Most tumors are difficult to detect in the early stages and are often discovered in the advanced stages, by which time metastasis has occurred (Bray et al., 2024). In recent years, cell therapy research of tumors, especially chimeric antigen receptor T-cell (CAR-T) therapy, has led to immuno-oncology in clinical trials (Tan et al., 2024). In addition, immune checkpoint therapy such as programmed death protein 1 (PD-1) and programmed cell death ligand 1 (PD-L1) has been applied in clinical practice for over a decade (Lin et al., 2024). These cell therapies have achieved outstanding success in tumor precision treatment. However, these therapies are hard to extend worldwide due to limitations and high treatment costs, making it urgent to find a specific biomarker for early diagnosis and prognosis of tumors.

CD248, a member of the C-type lectin domain (CTLD) group 14 family, is a single transmembrane glycoprotein (Khan et al., 2019; Barbera and Cucini, 2022). CD248 was once thought to be a marker of tumor endothelium and thus named endosialin or tumor endothelial marker 1 (TEM1), however, it is more recognized that CD248 is primarily expressed on stromal fibroblasts, pericytes, vascular smooth muscle cells, and perivascular mesenchymal stem cells, but not endothelial cells (Valdez et al., 2012; Payet et al., 2023; Kapopara et al., 2021). It has been reported that CD248 expression is substantially increased on tumor stroma in diverse cancers involving lung carcinoma, breast cancer, hepatocellular carcinoma, melanoma, and so on (Lu T. et al., 2023; Yang et al., 2020; Hong et al., 2022; D'Angelo et al., 2018). CD248 knockdown significantly suppresses tumor growth and metastasis of melanoma and hepatocellular carcinoma *in vivo* (Gan et al., 2024; Kuo et al., 2022). These findings imply that CD248 might serve as a potential oncogene and a promising candidate for targeted tumor therapies.

Clinically, high CD248 expression is associated with poor survival in cancer patients (Yang et al., 2020; Hong et al., 2022; Xiao et al., 2024). For example, Zeng et al. reveal that high CD248 expression is associated with poor prognosis and clinicopathological features such as lymphatic metastasis, tumor staging, and tumor-node metastasis (TNM) stage in non-small cell lung cancer (NSCLC) (Wu et al., 2022). In addition, CD248 upregulation is associated with poor prognosis of renal cell carcinoma (Xu et al., 2021), urothelial carcinoma (Li et al., 2021), hepatocellular carcinoma (Yang et al., 2020), colorectal cancer (Pietrzyk and Wdowiak, 2020), and so on. These findings suggest that CD248 might be a novel prognostic biomarker for tumors. There are several mechanisms by which CD248 regulates tumor growth and metastasis; for instance, Hsu et al. indicate that CD248 is involved in extracellular matrix (ECM) synthesis by activating the TGF-β signaling pathway (Hong et al., 2024); Zeng et al. demonstrate that CD248 promotes tumor progression by regulating immune cell infiltration or macrophage polarization (Wu et al., 2022). Huang et al. indicate that CD248 enhances tumor angiogenesis by upregulating two proangiogenic factors, osteopontin (OPN) and serpin family E member 1 (SERPINE1), via the Wnt/β-catenin signaling pathway (Hong et al., 2022). Taken together, CD248 might be a novel candidate for targeted therapies in pan-cancer. Pan-cancer and multi-omics research systematically analyze molecular features across cancer types, integrating genomics, transcriptomics, proteomics, and epigenomics. This approach uncovers shared mechanisms and cancer heterogeneity, thereby advancing basic cancer research and clinical translation (Gao et al., 2022; Anwaier et al., 2022). To the best of our knowledge, this is the first study to investigate the role of CD248 in pan-cancer, which provides new insights into its possible regulatory mechanisms and prognostic value. In this study, bioinformatic analysis and *in vitro* experiments were used to determine the effect of CD248 on pan-cancer. This study aimed to investigate the expression patterns, genetic alterations, and functional networks of CD248 in pan-cancer, to elucidate the relationship between CD248 expression and patient prognosis, and to detect CD248’s role in immune cell infiltration. The findings demonstrated that CD248 plays a crucial role in tumor growth and patient prognosis, indicating that CD248 could be an immunotherapeutic target for pan-cancer in the future.

## 2 Materials and methods

### 2.1 CD248 expression patterns in normal samples

CD248 mRNA and protein expression plots were conducted in the public Human Protein Atlas (HPA) Database (https://www.proteinatlas.org/). The expression of CD248 mRNA in different normal tissues was based on the Consensus dataset within the HPA Database, which is a transcriptome dataset of human normal tissues derived from RNA-seq data. CD248 protein expression in 44 tissues was based on knowledge-based annotation, with color coding based on tissue groups, each tissue group consists of tissues with common functional characteristics. CD248 mRNA expression in all single-cell types was a summary of normalized single-cell RNA (nTPM), and color-coding is based on cell type groups. Gene conservation analysis was conducted on the UCSC Genome Browser (https://genome.ucsc.edu/), comparing 11 typical vertebrate species, including mammals, birds, and fish. Immunohistochemical staining images of CD248 in several normal tissues and cancer samples were obtained from the public HPA Database.

### 2.2 CD248 protein expression in various cancer samples

The expression pattern of CD248 across diverse tumor tissues was analyzed in the GEPIA2 Database. The GEPIA2 Database was based on 9,736 tumors and 8,587 normal samples from the TCGA and the GTEx databases. GEPIA2 analysis concentrates on the expression of RNA sequencing, detailing 60,498 gene types and 198,619 isoform types. Data from 32 major tumor types were selected from the GEPIA2 database for CD248 expression analysis. Abbreviations and full names of 32 types of cancers in The Cancer Genome Atlas (TCGA) are shown in Table 1. The parameters set for differential gene expression analysis were |log2 fold change| > 1 and the P-value <0.05. The data from normal tissues were matched with both TCGA normal samples and GTEx data. The relationship between CD248 and cancer stage was analyzed by log_2_ (nTPM +1), and the threshold is P-value <0.05.

**TABLE 1:** Abbreviations and full names of 32 types of cancer used in this study.

Abbrev.	Full names
ACC	Adrenocortical carcinoma
BLCA	Bladder Urothelial carcinoma
BRCA	Breast invasive carcinoma
CESC	Cervical squamous cell carcinoma
CHOL	Cholangiocarcinoma
COAD	Colorectal adenocarcinoma
DLBC	Diffuse large B-cell lymphoma
GBM	Glioblastoma multiforme
ESCA	Esophageal carcinoma
HNSC	Head and neck squamous cell carcinoma
KICH	Kidney chromophobe
KIRC	Kidney renal clear cell carcinoma
KIRP	Kidney renal papillary cell carcinoma
LAML	Acute myeloid leukemia
LIHC	Liver hepatocellular carcinoma
LGG	Brain lower-grade glioma
LUSC	Lung squamous cell carcinoma
LUAD	Lung adenocarcinoma
MESO	Mesothelioma
OV	Ovarian serous cystadenocarcinoma
PAAD	Pancreatic adenocarcinoma
PCPG	Pheochromocytoma and paraganglioma
PRAD	Prostate adenocarcinoma
READ	Rectum adenocarcinoma
SARC	Sarcoma
SKCM	Skin cutaneous melanoma
STAD	Stomach adenocarcinoma
TGCT	Testicular Germ Cell Tumors
THCA	Thyroid carcinoma
UCEC	Uterine corpus endometrial carcinoma
UCS	Uterine carcinosarcoma
THYM	Thymoma

Notes: All abbreviations and their corresponding full names are consistent with those listed in TCGA Pan-Cancer Atlas.

### 2.3 The relationship between CD248 and patient prognosis

The relationship between CD248 and overall survival or disease-free survival of cancer patients was also analyzed in the GEPIA2 Database. All details of 32 types of cancers are shown in Table 1. The median was selected as the group cutoff, and the significance level is P-value <0.05. A hazard ratio <1 and P < 0.05 mean that a high expression level of the gene could increase the survival rate of the patient; a hazard ratio >1 and P < 0.05 mean that a high expression level of the gene could decrease the survival rate of the patients; a hazard ratio = 1 and P < 0.05 mean that the expression level of the gene is not connected with the survival rate of the patient.

### 2.4 The genetic alteration of CD248 in various tumors

Genetic alteration of CD248 in 32 kinds of tumors was analyzed from the cBioPortal Database (https://www.cbioportal.org/). Mutation counts of CD248 in several cancers were also analyzed in the cBioPortal Database. The relationship between CD248 mutations and patient survival was analyzed by the log-rank test P-value, and the threshold is P-value <0.05.

### 2.5 The relationship between CD248 expression and immune infiltrates

The association between CD248 expression and immune infiltrates in 40 types of cancers was attained from the TIMER 2.0 Database (http://timer.cistrome.org/). The heatmap shows the purity-adjusted Spearman’s rho across various cancer types (red presents a positive association, and blue represents a negative association). The estimations from EPIC, MCPCOUNTER, XCELL, and TIDE algorithms were applied to analyze the relationship between CD248 and immune cell infiltration, including cancer-associated fibroblasts (CAFs), macrophages, monocytes, CD4^+^ T cells, CD8^+^ T cells, and Tregs.

### 2.6 CD248-related gene analysis in pan-cancer

Protein-protein interaction (PPI) of CD248 was analyzed through the STRING tool (https://cn.string-db.org/). The minimum required interaction score was set as medium confidence (0.4). The line thickness of network edges indicated the strength of data support. The relationship between CD248 and proteins in the network across all tumor samples from TCGA was analyzed in the GEPIA2 Database, and then the top seven related proteins were listed. The interactions between CD248 and those seven proteins were also predicted using AlphaFold3 (https://alphafoldserver.com/) and subsequently visualized with PyMOL software. CD248-protein interactions were also identified through the BioGRID Database, based on published studies (https://thebiogrid.org/). Expression profiles of CD248 in HNSC were retrieved from the TCGA database, followed by Gene Ontology (GO) enrichment analysis and Kyoto Encyclopedia of Genes and Genomes (KEGG) pathway enrichment analysis to elucidate the biological functions and pathways associated with CD248.

### 2.7 CD248 expression in tumor and stromal cells analyzed by single-cell sequencing

Correlation between CD248 expression and TMB or MSI was conducted in the bioinformatics database (https://www.bioinformatics.com.cn/). CD248 expression in tumor and stromal cells was analyzed by single-cell sequencing in the Cancer SCEM Database (https://ngdc.cncb.ac.cn/), and CD248 expression level in single cells of tumor samples was visualized through UMAP.

### 2.8 Cell culture and transfection of small interfering RNA (siRNA)

Human oral keratinocytes (HOK), human squamous cell carcinoma 3 (HSC3), human nasopharyngeal carcinoma 6 (HN6), human squamous cell carcinoma of the head and neck 27 (CAL27), and mouse squamous cell carcinoma 7 (SCC-7) were cultured with DMEM medium (Gibco) comprising 10% (v/v) fetal bovine serum (FBS) and 1% (v/v) penicillin/streptomycin solution in a 37°C, 5% CO_2_ incubator. All cell lines present in this study were purchased from Zhong Qiao Xin Zhou Biotech (Shanghai, China) and verified by our colleges (Yang et al., 2024; Li et al., 2024). As for the transfection of siCD248, the cells were plated in 6-well plates, and 5 µL/well siCD248 (final concentration of 50 nM) in 250 µL Opti-MEM (Gibco) was prepared and combined with 5 µL Lipofectamine 3,000 (Thermo Fisher) diluted in 250 µL Opti-MEM. After a 15-min incubation at room temperature, the siRNA-Lipofectamine complex was added to the cells and incubated for 6 h in a 37°C incubator. The medium was then removed and replaced with new culture medium, and the cells were further cultured for 24 h. Western blot assays were performed to evaluate the silencing effect of siCD248. The sequence of siCD248 is shown in Supplementary Table S1.

### 2.9 Quantitative real-time polymerase chain reaction (qRT-PCR)

Total RNA was isolated from cells with an RNA isolation kit with spin columns (Beyotime). 1,000 ng RNA was used for the reverse transcription process to generate cDNA using the Evo M-MLV RT Kit (AG Biology). qRT-PCR was performed with 2x SP qPCR Mix (BG Biotech) on a CFX96 Real-Time PCR Detection System (Bio-Rad). mRNA expressions of CD248 were measured, and GAPDH was used as a reference gene. The primer sequences are shown in Supplementary Table S2.

### 2.10 Western blot

Cells were lysed with RIPA lysis buffer (Beyotime) consisting of 1 mM protease inhibitor cocktail (Beyotime). Protein concentrations were quantified using a BCA protein assay kit (Beyotime). Denaturation of all samples was performed at 100°C for 10 min. Proteins in equal amounts (25 μg in total) were separated by SDS-PAGE (Epizyme) and then transferred to Immobilon®-FL transfer membranes (Millipore). After blocking step with 5% nonfat milk for 2 h, membranes were incubated with primary antibodies against CD248 (#47948, CST) and β-actin (#4967, CST) (1:1,000) at 4°C overnight, respectively. After that, membranes were incubated with HRP-tagged goat anti-rabbit IgG (1: 3000, CST) for 1 h. Protein bands were visualized with the BeyoECL Plus kit (Beyotime), followed by quantification of band intensities with ImageJ software.

### 2.11 Transwell assay

Cell invasion was tested by transwell assay. After siCD248 treatment for 24 h, a total of 10^4^ cells were cultured on the upper surface of the transwell insert. After 24 h, the insert was fixed with methanol, stained with 0.1% crystal violet for 15 min, and rinsed several times. Cells on the upper surface of the insert were carefully removed. Five random fields on the lower surface of the insert were photographed under a microscope and analyzed by ImageJ.

### 2.12 Scratch wound healing assay

Scratch wound healing assay was used to evaluate cell migration. Cells were cultured in a 6-well plate and transfected with siCD248 treatment for 24 h. After that, scratches were made across each well with an aseptic 200 μL micropipette tip, and floating cells were washed with PBS. Scratch images were taken at 0 h and 24 h under a microscope and analyzed by ImageJ.

### 2.13 EdU proliferating assay

Cell proliferation was evaluated using the kFluor488 Click-iT EdU Kit (Keygen). After siCD248 treatment for 24 h, a total of 2 × 10^4^ cells were cultured in a 96-well plate. After 24 h, 2x EdU working solution was added to the cells and incubated for 1 h. Cells were then fixed with 4% paraformaldehyde for 15 min and stained with the Click-iT working solution for 30 min. Five random fields were photographed under a microscope and analyzed by ImageJ.

### 2.14 Statistical analysis

Statistical analysis was performed using GraphPad Prism (version 8.0), and data were analyzed for statistical significance using one-way ANOVA analysis. Statistical significance was determined with a threshold of P < 0.05. Experiments were conducted independently three times, and data are shown as mean (SD).

## 3 Results

### 3.1 CD248 expression profile in normal tissues and cells

**FIGURE 1:**
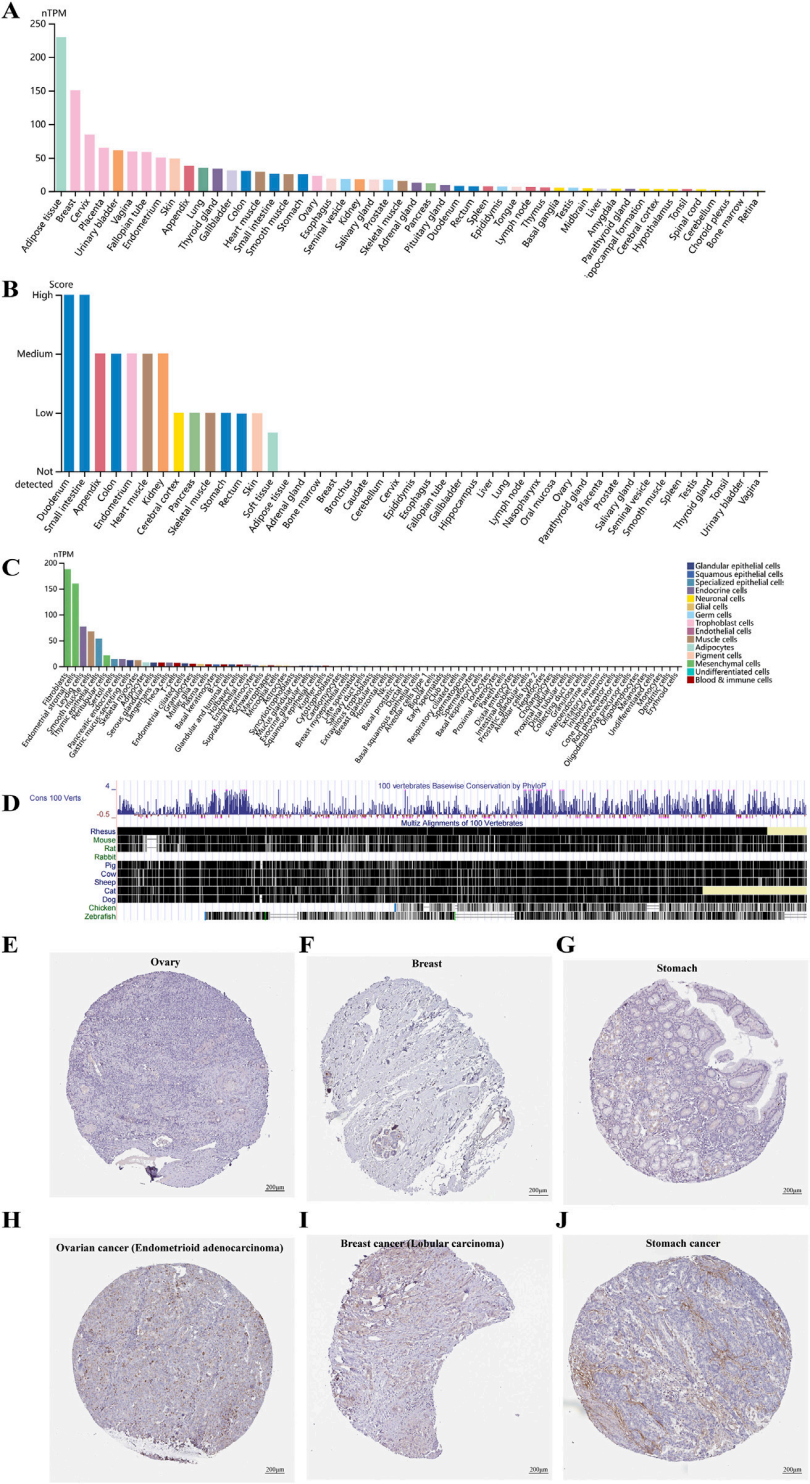
CD248 expression profile in different tissues and cells **(A)** The expression levels of CD248 mRNA in diverse tissues. **(B)** The expression levels of CD248 protein in various tissues **(C)** Single-cell type specificity of CD248 mRNA. **(D)** Gene conservation of CD248 in different vertebrates **(E–G)** Immunohistochemical staining of CD248 protein in normal ovary, breast, and stomach tissue, respectively. Scale bar = 200 μm. **(H–J)** Immunohistochemical staining of CD248 protein in ovary cancer (endometrioid carcinoma subtype), breast cancer (lobular carcinoma subtype), and stomach cancer, respectively. nTPM, normalized Transcripts Per Million. All data are from the HPA Database. Scale bar = 200 μm.

Based on the HPA Database, we found that CD248 mRNA was differentially expressed in 50 types of normal tissues, with high expression in the top 10% of tissues, including adipose, breast, cervix, placenta, and urinary bladder (Figure 1A). Similarly, CD248 protein was differentially expressed in 14 types of normal tissues, with high expression in the top 10% of tissues, including the duodenum and small intestine. Whereas CD248 protein expression was undetected in 31 other tissues (Figure 1B). Single-cell specificity indicated that CD248 mRNA was differentially expressed in 53 types of normal cells, with high expression in the top 5% of cells, including fibroblasts, endometrial stromal cells, Leydig cells, and smooth muscle cells. While CD248 mRNA expression was not found in 26 types of normal cells (Figure 1C). In summary, CD248 is mostly expressed in tissues enriched with mesenchymal cells and epithelial cells. Cross-species conservation revealed that CD248 was highly conserved in various vertebrates, including primates (rhesus), euarchontoglires (mouse, rat), and laurasiatheria (dog, cat, pig, sheep, and cow), while CD248 was not completely conserved in birds (chickens) and fish (zebrafish) (Figure 1D). Immunohistochemical staining indicated that CD248 protein was undetectable in normal ovary, breast, and stomach tissues (Figures 1E-G), consistent with Figure 1B findings.

### 3.2 CD248 expression profile in various cancer samples

**FIGURE 2:**
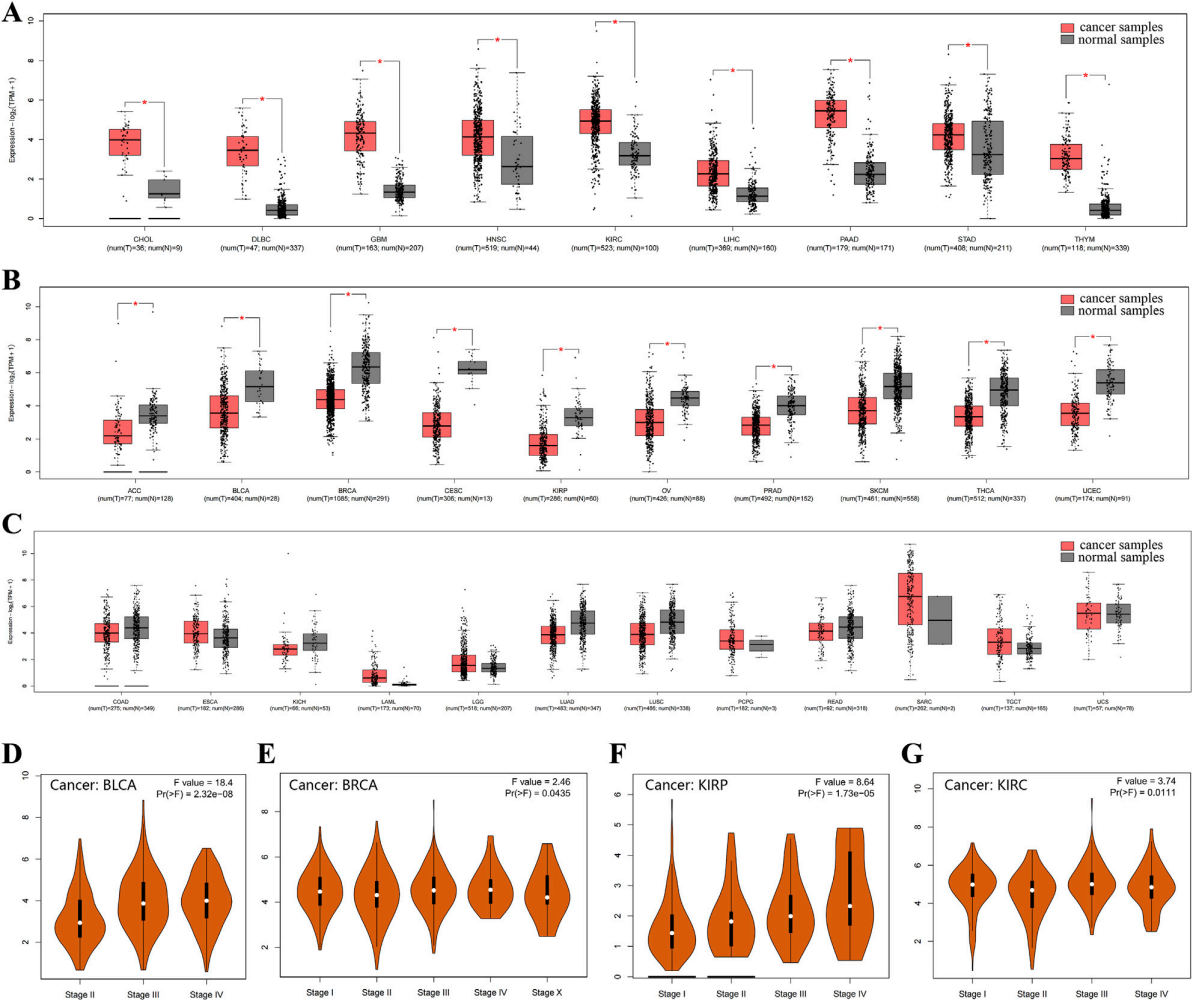
CD248 mRNA expression in various cancer and control samples **(A)** CD248 mRNA expression was increased in nine types of cancers. **(B)** CD248 mRNA expression was decreased in ten kinds of cancers. **(C)** There was no significant difference in CD248 mRNA expression in twelve types of cancer. **(D-G)** Violin plots of cancer staging in BLCA **(D)**, BRCA **(E)**, KIRP **(F)**, and KIRC **(G)**, respectively. *P < 0.05. All data are from the GEPIA2 Database.

Immunohistochemical staining indicated that CD248 protein expression was upregulated in endometrioid carcinoma subtype of ovarian cancer (Figure 1H), in breast lobular carcinoma subtype of breast cancer (Figure 1I), as well as in stomach adenocarcinoma (Figure 1J). The expression pattern of CD248 mRNA across diverse tumor tissues was analyzed in the GEPIA2 Database. The results indicated that CD248 mRNA level was increased in nine types of tumor samples compared to normal tissues, including CHOL, DLBC, GBM, HNSC, KIRC, LIHC, PAAD, STAD, and THYM (Figure 2A, P < 0.05). Conversely, CD248 mRNA level was decreased in ten types of tumor samples compared to normal controls, such as ACC, BLCA, BRCA, CESC, KIRP, OV, PRAD, SKCM, THCA, and UCEC (Figure 2B, P < 0.05). In addition, CD248 mRNA level was not significantly different in twelve types of cancers compared to the control group, including COAD, ESCA, KICH, LAML, LGG, LUAD, LUSC, PCPG, READ, SARC, TGCT, and UCS (Figure 2C, P > 0.05). In the violin plot of cancer staging, high expression of CD248 was significantly correlated with advanced stages of BLCA (P < 0.05, Figure 2D), BRCA (P < 0.05, Figure 2E), KIRP (P < 0.05, Figure 2F), and KIRC (P < 0.05, Figure 2G). Taken together, CD248 expression was significantly upregulated in several types of cancer, suggesting its potential role in cancer diagnosis and therapy.

### 3.3 The relationship between CD248 and patient prognosis

**FIGURE 3:**
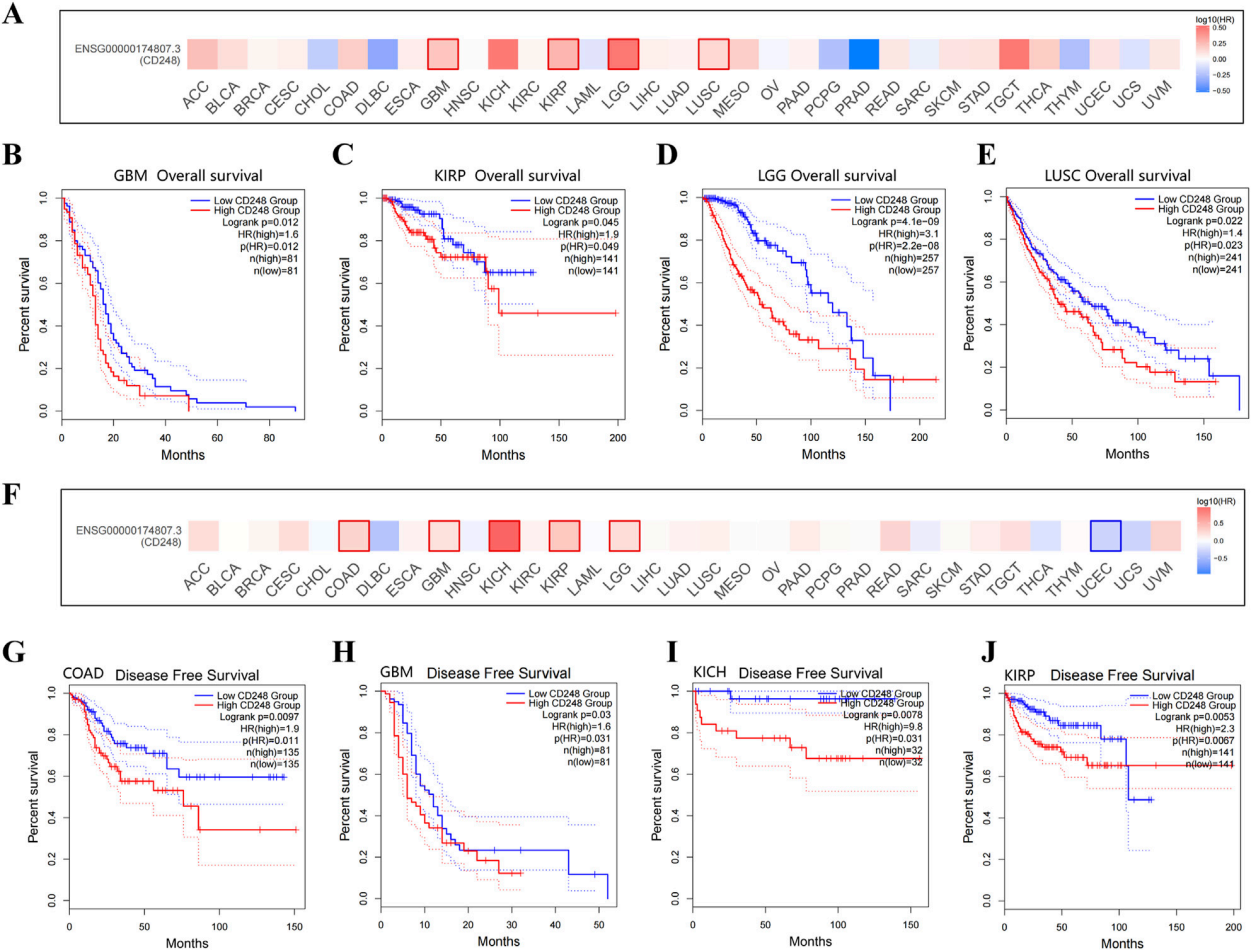
The relationship between CD248 expression and patient prognosis **(A)** Correlation between CD248 expression and overall survival in multiple cancers. **(B–E)** The difference in the overall survival between high (red line) and low CD248 expression groups (blue line) in GBM, KIRP, LGG, and LUSC. **(F)** Correlation between the expression of CD248 and disease-free survival in multiple cancers. **(G–J)** The difference in the disease-free survival between high (red line) and low CD248 expression groups (blue line) in COAD, GBM, KICH, and KIRP. **(G-J)** The difference in the disease-free survival between high (red line) and low CD248 expression groups (blue line) in COAD, GBM, KICH, and KIRP, respectively. See Supplementary Figures S1A, B for disease-free survival in LGG and UCEC. All data are from the GEPIA2 Database.

The relationship between CD248 expression and patient prognosis was analyzed in the GEPIA2 Database. High expression of CD248 was significantly associated with poor overall survival in several types of cancer (Figure 3A), including GBM (HR = 1.6, P = 0.012) (Figure 3B), KIRP (HR = 1.9, P = 0.049) (Figure 3C), LGG (HR = 3.1, P < 0.001) (Figure 3D), and LUSC (HR = 1.4, P = 0.023) (Figure 3E). In addition, high expression of CD248 was significantly associated with poor disease-free survival in several types of cancer (Figure 3F), including COAD (HR = 1.9, P = 0.011) (Figure 3G), GBM (HR = 1.6, P = 0.031) (Figure 3H), KICH (HR = 9.8, P = 0.031) (Figure 3I), KIRP (HR = 2.3, P = 0.0067) (Figure 3J), and LGG (HR = 1.6, P = 0.012) (Supplementary Figure S1A), while high expression of CD248 was negatively related to the disease-free survival of UCEC (HR = 0.5, P = 0.046) (Supplementary Figure S1B). Taken together, high CD248 expression was unfavorable in GBM, KIRP, and LGG, suggesting CD248 is a potential prognostic indicator.

### 3.4 The genetic alteration of CD248 in various tumors

**FIGURE 4:**
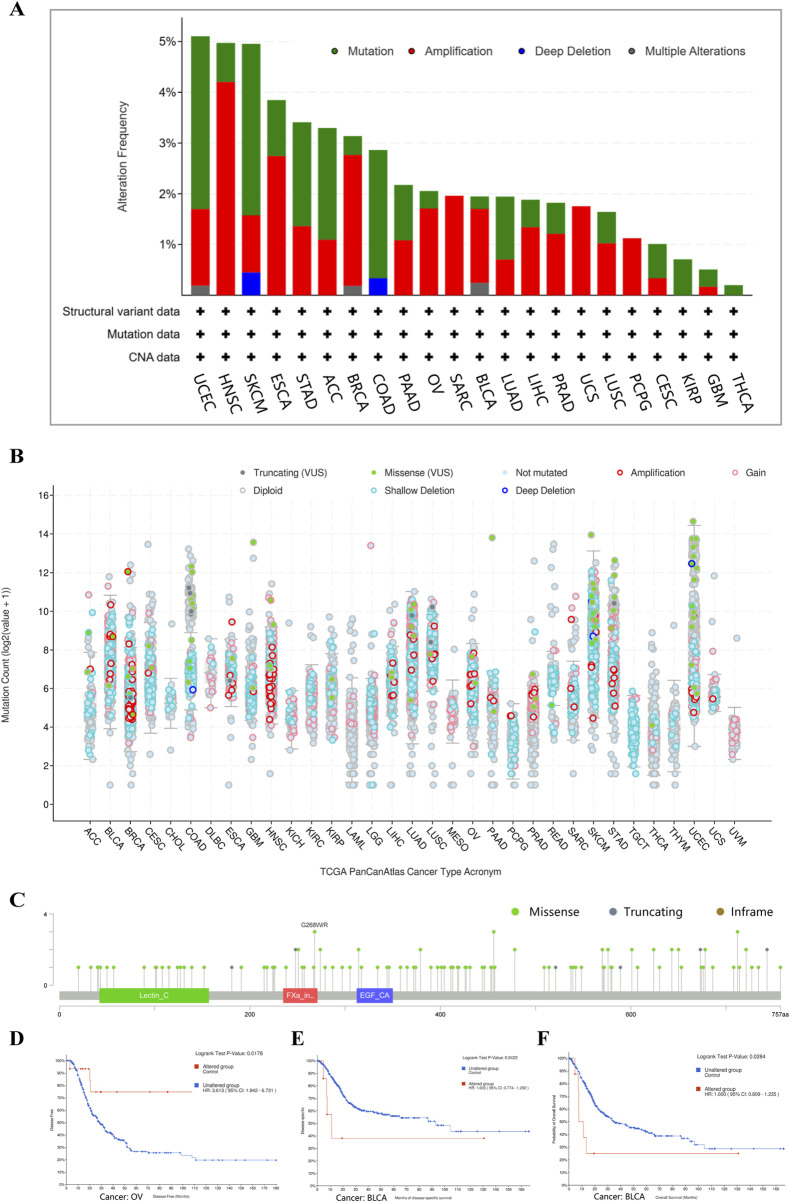
The genetic alterations of CD248 in various tumors in TCGA datasets **(A)** Four types of gene alterations of CD248 in several TCGA tumors. **(B)** Mutation counts of CD248 in several cancers. **(C)** Mutation sites of CD248 in several TCGA tumors. **(D)** The relationship between CD248 mutation and OV disease-free survival. **(E, F)** The relationships between CD248 mutation and both disease-free survival **(E)** and overall survival **(F)** in BLCA. All data were from the cBioPortal Database.

The genetic alterations of CD248 across different tumor types within the TCGA datasets were studied using cBioPortal. Mutations and amplifications represented the predominant form of CD248 genetic alterations in TCGA tumor samples. The mutation and amplification frequency were highest within UCEC, HNSC, and SKCM, exceeding 4% (Figures 4A, B). CD248 genetic alterations were not observed in 10 types of tumors, including LAML, GBM, CHOL, DLBC, KICH, KIRC, MESO, TGCT, THYM, and UVM (data are not shown). A total of 212 mutation sites of CD248 had been identified, comprising 155 missense mutations, 55 truncating mutations, and two inframe mutations (Figure 4C). CD248 mutation was linked to better prognosis in OV patients in terms of disease-free survival (P = 0.0178) (Figure 4D), whereas CD248 mutation was linked to poor prognosis in BLCA patients in terms of disease-free survival (P = 0.0422) (Figure 4E) and overall survival (P = 0.0284) (Figure 4F).

### 3.5 The relationship between CD248 expression and immune cell infiltration

**FIGURE 5:**
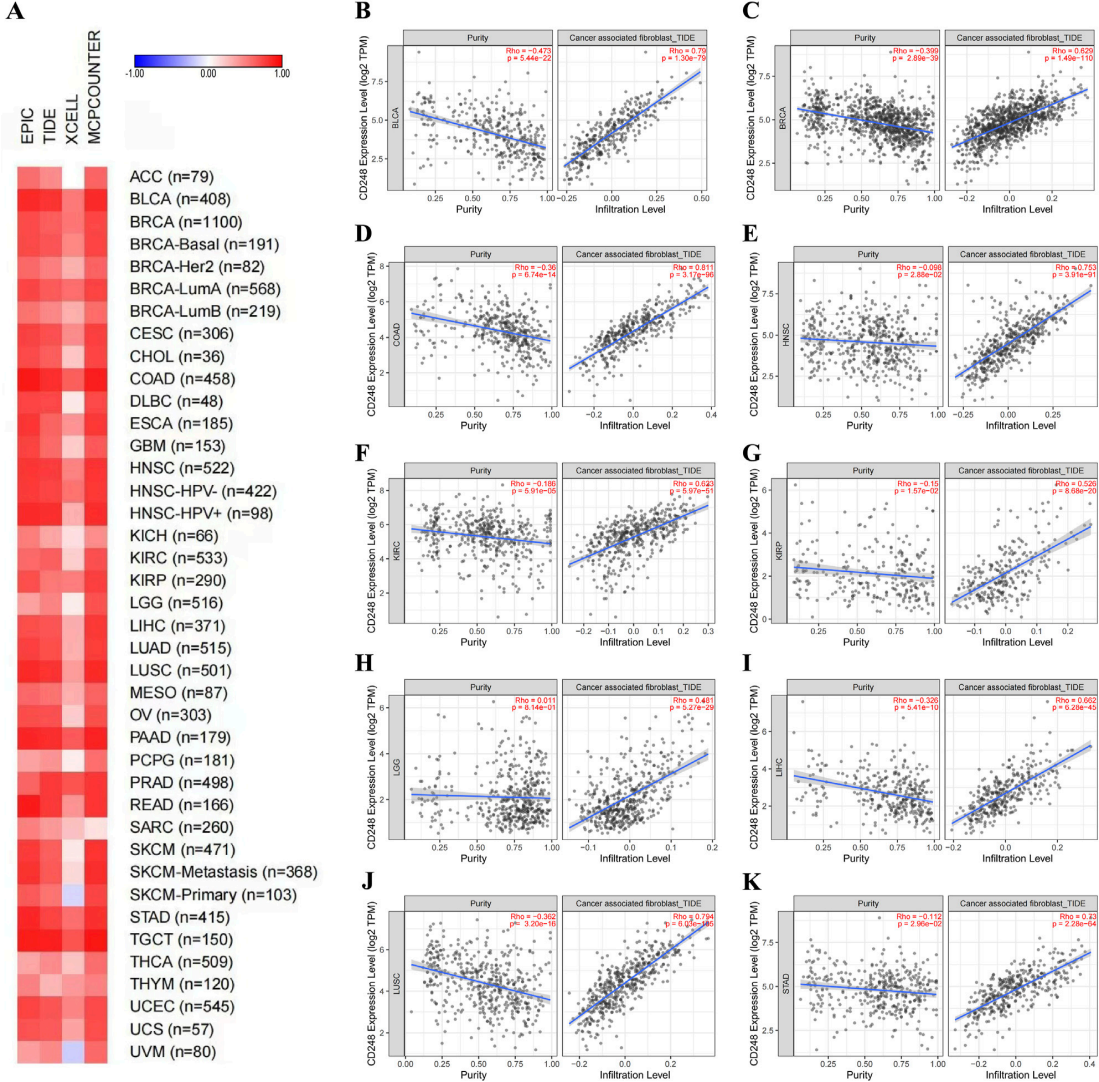
The relationship between CD248 expression and CAFs infiltration **(A)** The relationship between CD248 expression and CAFs infiltration in 40 types of cancers. **(B–K)** The relationship between CD248 expression and CAFs infiltration in BLCA, BRCA, COAD, HNSC, KIRC, KIRP, LGG, LIHC, LUSC, and STAD. See Supplementary Figures S2A–F for CAFs infiltration in GBM, HNSC (HPV-), HNSC (HPV+), OV, PAAD, and THYM. All data are from the TIMER 2.0 database.

**FIGURE 6:**
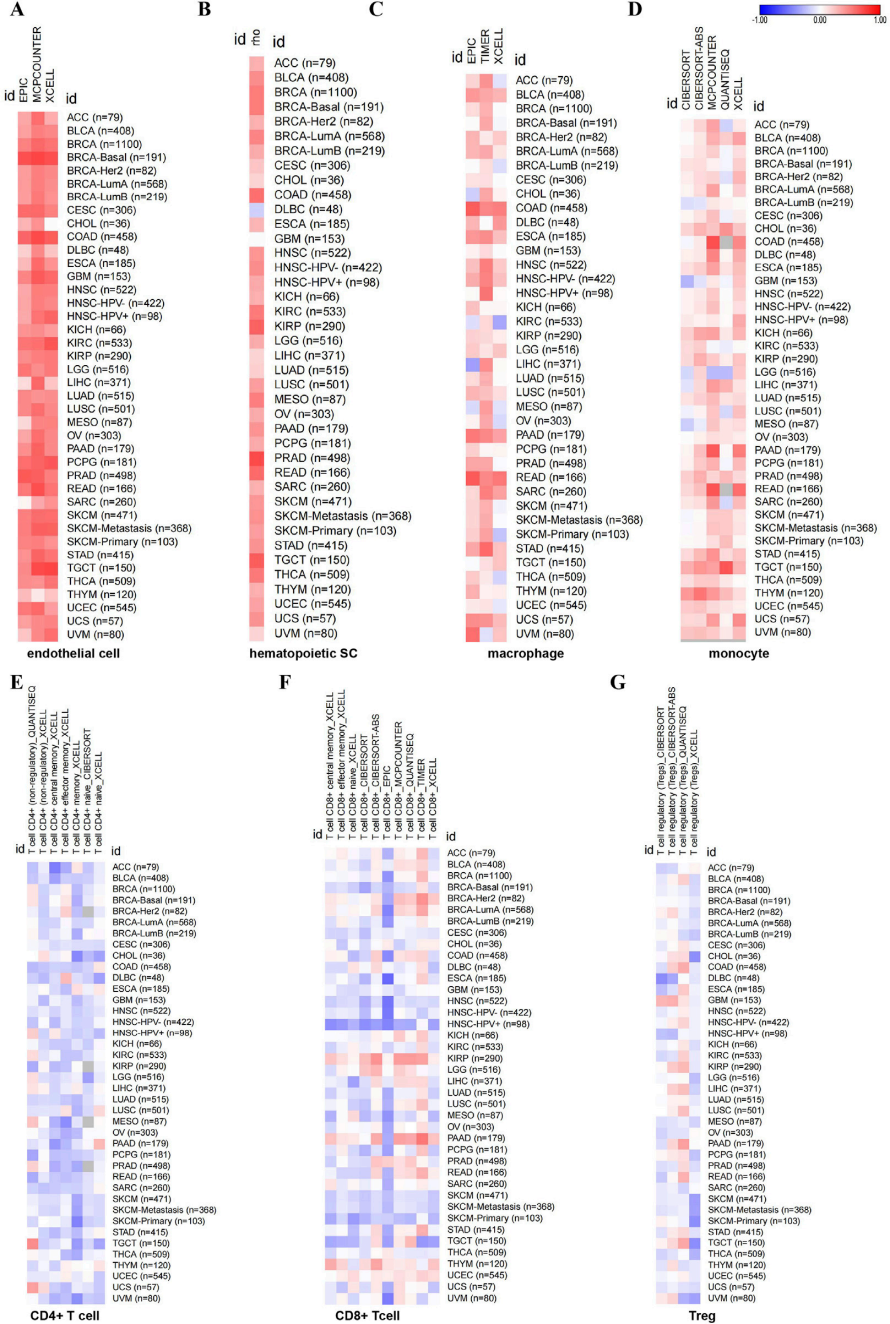
The relationship between CD248 expression and immune cell infiltration in pan-cancer **(A–G)** The relationship between CD248 expression and endothelial cell infiltration **(A)**, hematopoietic stem cell infiltration **(B)**, macrophage infiltration **(C)**, monocyte infiltration **(D)**, CD4^+^ T cell infiltration **(E)**, CD8^+^ T cell **(F)**, and Treg infiltration **(G)**, respectively. All data are from the TIMER 2.0 database.

Some studies have shown that CD248 is mainly expressed in fibroblasts. Hence, we investigated the relationship between CD248 expression and cancer-associated fibroblasts (CAFs) infiltration in several cancers. EPIC and TIDE algorithms showed that CD248 was correlated with CAFs infiltration in 40 types of cancers (Figure 5A). Beyond that, the results of TIDE algorithms showed that CD248 expression was positively correlated with CAFs infiltration in various cancers, including BLCA (Rho = 0.79), BRCA (Rho = 0.629), COAD (Rho = 0.811), HNSC (Rho = 0.753), KIRC (Rho = 0.623), KIRP (Rho = 0.526), LGG (Rho = 0.481), LIHC (Rho = 0.662), LUSC (Rho = 0.794), STAD (Rho = 0.73) (Figures 5B–K), GBM (Rho = 0.584), HNSC (HPV-) (Rho = 0.726), HNSC (HPV+) (Rho = 0.806), OV (Rho = 0.679), PAAD (Rho = 0.818), and THYM (Rho = 0.287) (Supplementary Figures S2A–F) (P < 0.001). Particularly, CD248 was positively related to CAFs infiltration in both HPV-negative and HPV-positive HNSC. In addition, CD248 expression was associated with endothelial cell and hematopoietic stem cell infiltration, suggesting that CD248 was likely linked to angiogenesis in the tumor microenvironment (TME) (Figures 6A, B). CD248 was also positively associated with infiltration of immune cells, such as macrophages and monocytes (Figures 6C, D), however, CD248 was negatively related to CD4^+^ T cell, CD8^+^ T cell, and Treg infiltration in tumors (Figures 6E–G).

### 3.6 CD248-related gene analysis in pan-cancer

**FIGURE 7:**
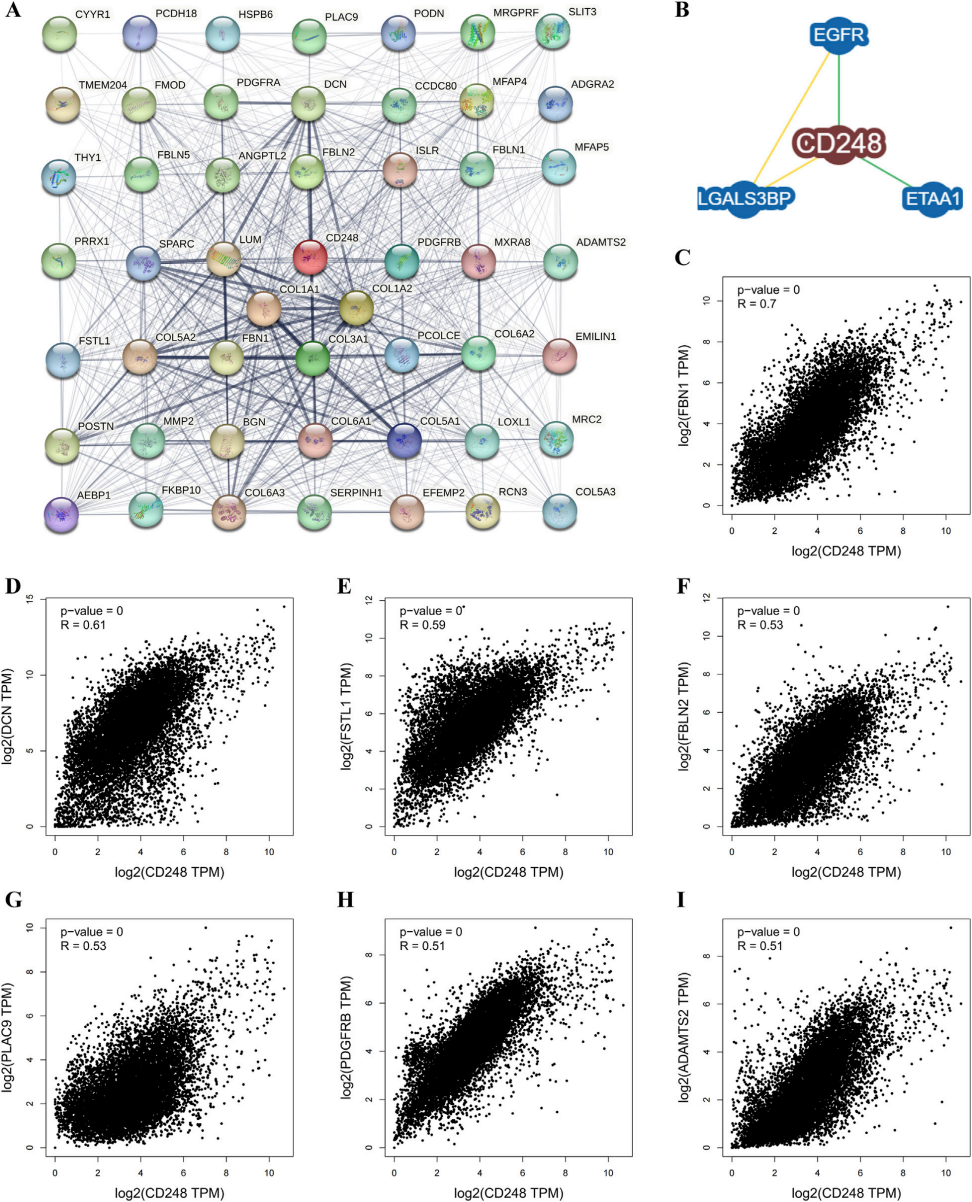
CD248-related gene analysis in pan-cancer **(A)** Protein-protein interaction (PPI) network of CD248 by the STRING tool. **(B)** CD248-protein interactions obtained by the BioGRID Database. **(C–I)** Interaction analysis between CD248 and **(C)** FBN1, **(D)** DCN, **(E)** FSTL1, **(F)** FBLN2, **(G)** PLAC9, **(H)** PDGFRB, and **(I)** ADAMTS2 across all tumor samples from the GEPIA2 Database.

To find out the mechanism by which CD248 mediates the growth of tumors, CD248-related proteins were analyzed using the STRING tool. Among 50 proteins interacting with CD248, there were 6 interacting proteins with medium confidence, including collagen α-1(I) chain (COL1A1) (score = 0.525), collagen α-2(I) chain (COL1A2) (score = 0.509), fibulin-2 (FBLN2) (score = 0.469), collagen α-1(III) chain (COL3A1) (score = 0.453), collagen α-2(VI) chain (COL6A2) (score = 0.452), and platelet-derived growth factor receptor beta (PDGFRB) (score = 0.419) (Figure 7A; Supplementary Table S3). Besides that, BioGRID analysis showed that CD248 interacted with epidermal growth factor receptor (EGFR) (Zeng et al., 2019), ewing tumor-associated antigen 1 (ETAA1) (Achuthankutty et al., 2019), galectin three binding protein (LGALS3BP) (Figure 7B) (Becker et al., 2008). Furthermore, correlation analysis between CD248 and those interacted proteins across all tumor samples from TCGA was conducted in the GEPIA2 database, the results showed that CD248 was positively correlated with fibrillin 1 (FBN1, R = 0.7) (Figure 7C), decorin (DCN, R = 0.61) (Figure 7D), follistatin-like 1 (FSTL1, R = 0.59) (Figure 7E), fibulin 2 (FBLN2, R = 0.53) (Figure 7F), placenta-associated 9 (PLAC9, R = 0.53) (Figure 7G), platelet-derived growth factor receptor beta (PDGFRB, R = 0.51) (Figure 7H), and ADAM metallopeptidase with thrombospondin type 1 motif 2 (ADAMTS2, R = 0.51) (Figure 7I).

**FIGURE 8:**
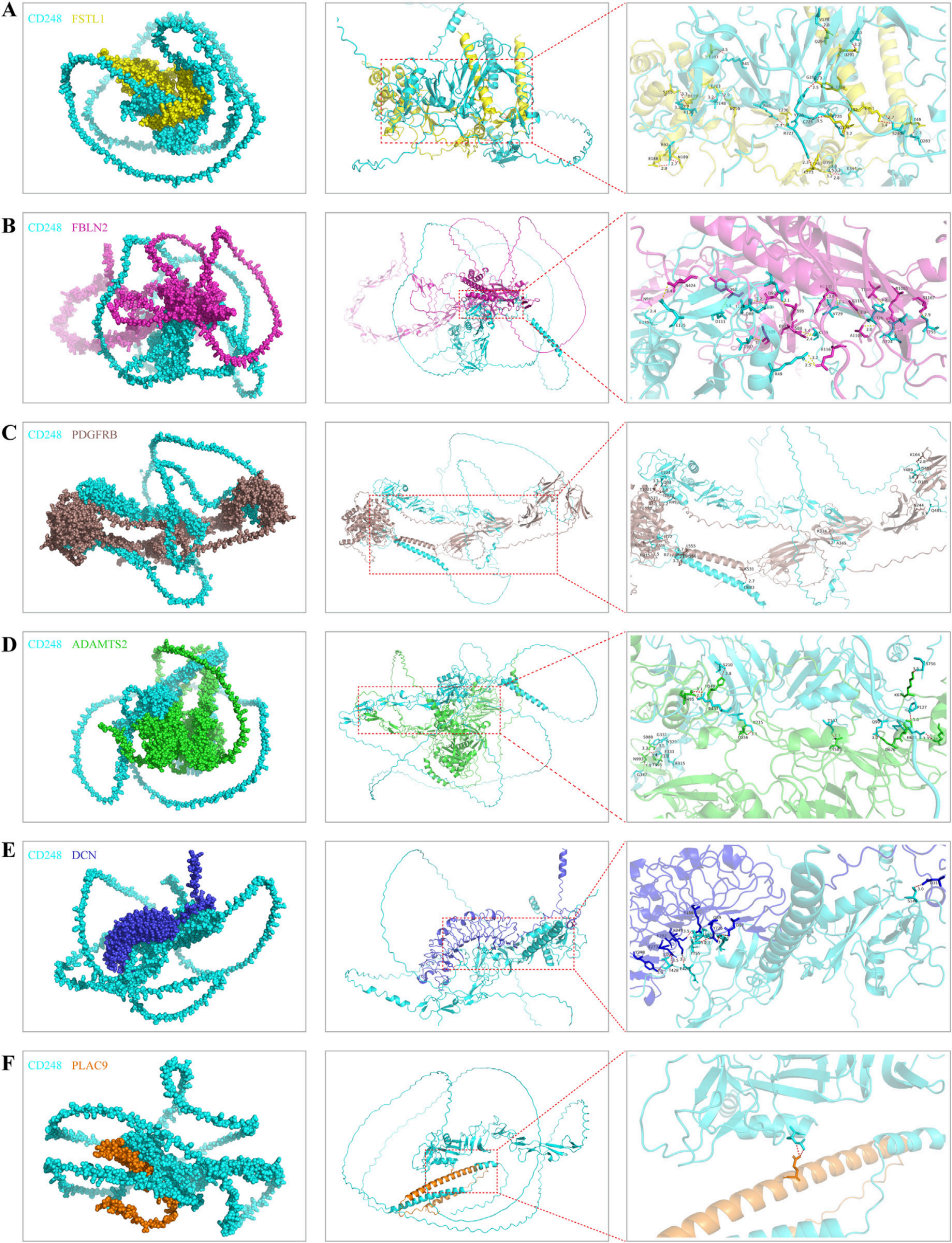
The predicted interaction diagrams between CD248 and the other interacted proteins **(A–F)** The predicted interaction diagrams between CD248 and **(A)** FSTL1, **(B)** FBLN2, **(C)** PDGFRB, **(D)** ADAMTS2, **(E)** DCN, **(F)** PLAC9. The red dashed lines in the right column figures are hydrogen bonds. All diagrams were conducted by AlphaFold3 and PyMOL software.

Using AlphaFold3 and PyMOL, we predicted putative interactions between CD248 and the above seven proteins. The number of hydrogen bonds formed between CD248 and each protein was as follows: 24 hydrogen bonds with FSTL1 (Figure 8A), 19 hydrogen bonds with FBLN2 (Figure 8B), 15 hydrogen bonds with PDGFRB (Figure 8C), and 14 hydrogen bonds with ADAMTS2 (Figure 8D), 11 hydrogen bonds with DCN (Figure 8E), 2 hydrogen bonds with PLAC9 (Figure 8F), and 0 hydrogen bonds with FBN1 (data not shown). In addition, CD248 was predicted to interact with CD93 (Supplementary Figure S3A) and CLEC14A (Supplementary Figure S3B), with 8 hydrogen bonds in each interaction. Furthermore, we predicted potential interactions between CD248 and 12 common tumor biomarkers, with varying hydrogen bond counts formed between CD248 and each protein: 40 hydrogen bonds with cancer antigen 153 (CA153, Supplementary Figure S4A); 15 hydrogen bonds with prostate-specific Antigen (PSA, Supplementary Figure S4B); 10 hydrogen bonds with squamous cell carcinoma antigen 1 (SCCA1, Supplementary Figure S4C), and alpha-fetoprotein (AFP) (Supplementary Figure S4D); 9 hydrogen bonds with carbohydrate antigen 19–9 (CA19-9, Supplementary Figure S4E), carcinoembryonic antigen (CEA, Supplementary Figure S4F), and squamous cell carcinoma antigen 2 (SCCA2, Supplementary Figure S5A); 8 hydrogen bonds with programmed cell death ligand 1 (PD-L1, Supplementary Figure S5B); 7 hydrogen bonds with neuron-specific enolase (NSE, Supplementary Figure S5C), and gastrin-releasing peptide (GRP) (and Supplementary Figure S5D); 5 hydrogen bonds with cytokeratin 19 fragment (CYFRA21-1, Supplementary Figure S5E); none with cancer antigen 50 (CA50, data not shown). All these suggest the potential target therapy of CD248 in pan-cancer.

### 3.7 CD248 expression in tumor and stromal cells analyzed by single-cell sequencing

**FIGURE 9:**
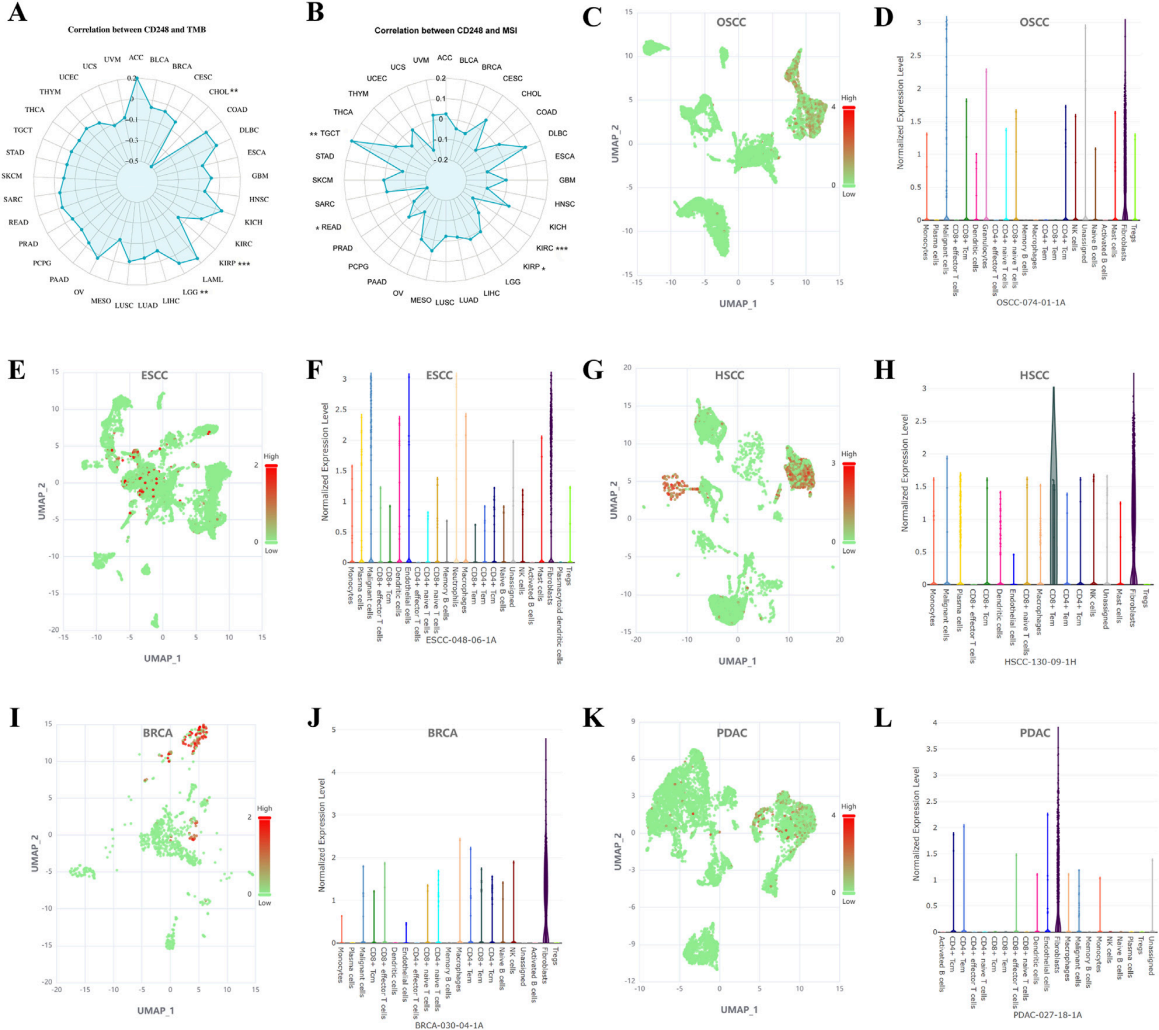
CD248 expression in tumor and stromal cells was analyzed by single-cell sequencing **(A)** Correlation between CD248 expression and TMB. *P < 0.05; **P < 0.01; ***P < 0.001. **(B)** Correlation between CD248 expression and MSI. *P < 0.05; **P < 0.01; ***P < 0.001 **(C–L)** Single-cell sequencing analyzing the co-expression of CD248 on tumor and stromal cells in OSCC **(C, D)**, ESCC **(E, F)**, HSCC **(G, H)**, BRCA **(I, J)**, and PDAC **(K, L)**. red means high gene expression, and green means low gene expression. See Supplementary Figures S6A–H for single-cell sequencing in PAAD, GBM, CRC, and SKCM. All data are from the Cancer SCEM database.

CD248 expression was positively related to tumor mutation burden (TMB) in LGG (P = 0.0043), while negatively related to TMB in KIRP (P = 0.0009) and CHOL (P = 0.0028) (Figure 9A; Supplementary Table S4). In addition, CD248 expression was positively related to microsatellite instability (MSI) in TGCT (P = 0.0072) while negatively related to MSI in KIRC (P = 0.0008), READ (P = 0.0168), and KIRP (P = 0.0215) (Figure 9B; Supplementary Table S5). Then, single-cell sequencing revealed that CD248 is co-expressed on tumor and stromal cells within the TME in various cancers, including OSCC (Figures 9C, D), ESCC (Figures 9E, F), HSCC (Figures 9G, H), BRCA (Figures 9I, J), PDAC (Figures 9K, L), PAAD (Supplementary Figures S6A, B), GBM (Supplementary Figures S6C, D), CRC (Supplementary Figures S6E, F), and SKCM (Supplementary Figures S6G, H). In addition, CD248 is co-expressed on tumor and stromal cells, such as fibroblasts, CD4^+^ central memory T cells (CD4^+^ Tcm), CD4^+^ effector memory T cells (CD4^+^ Tem), CD8^+^ Tem, CD8^+^ Tcm, CD8^+^ effector T cells, dendritic cells, endothelial cells, granulocytes, macrophages, malignant cells, neutrophils, plasma cells, and smooth muscle cells, suggesting a potential significant role of CD248 in cancer therapy and prognosis (Figures 9C–L; Supplementary Figures S6A–H).

### 3.8 The role of CD248 in HNSC cell lines

**FIGURE 10:**
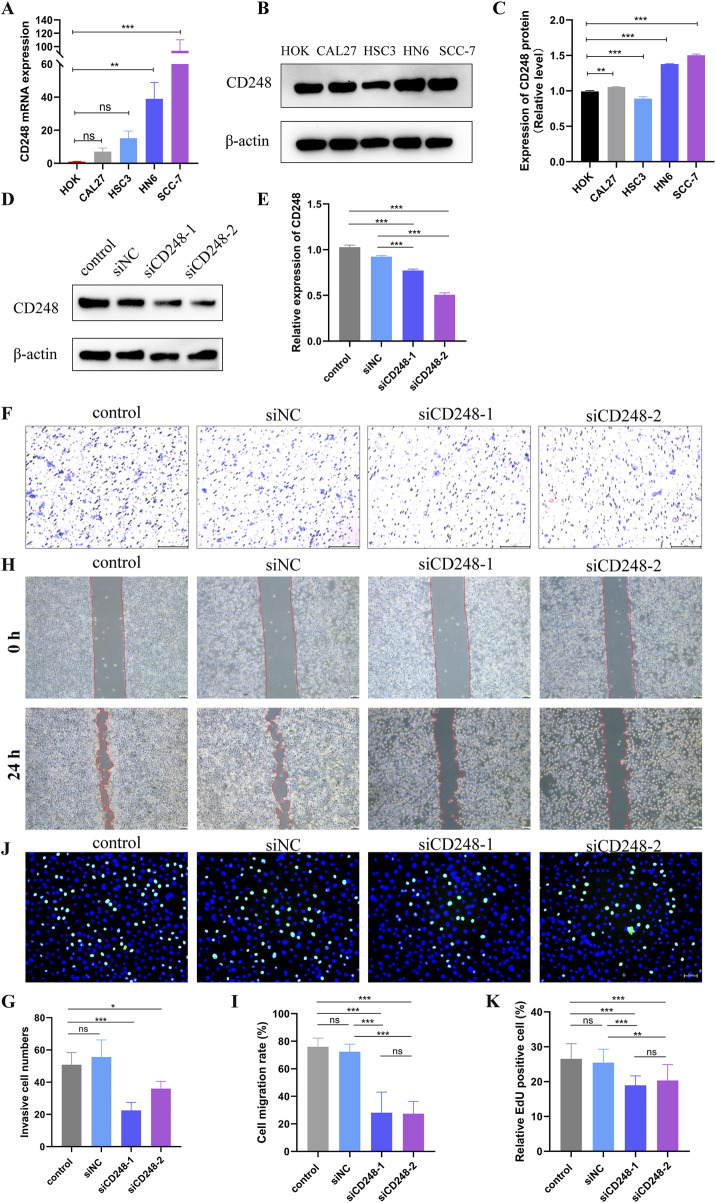
The role of CD248 in HNSC cell lines **(A)** CD248 mRNA expression in HNSC cell lines. **(B)** CD248 protein expression in HNSC cell lines. **(C)** Relative expression of CD248 in **(B)** after normalization to β-actin. Uncropped blots are presented in Supplementary Figure S7. **(D)** CD248 protein expression in HN6 cells after treatment with 50 nM siCD248 for 48 h. Uncropped blots are presented in Supplementary Figure S7 **(E)** Relative CD248 expression in **(D)** after normalization to that of β-actin. **(F)** The invasion of HN6 cells by transwell assay after 50 nM siCD248 treatment. Scale bar = 200 μm. **(G)** Statistical analysis of the number of invasive cells. **(H)** Scratch assays of HN6 cells at 0 h and 24 h after siCD248 treatment. Scale bar = 100 μm. **(I)** Quantitative analysis of the cell migration rate **(J)** EdU assay to detect the proliferation of HN6 cells. Blue is DAPI, and green represents proliferating cells. Scale bar = 50 μm. **(K)** The proportion of proliferative HN6 cells in **(J)**. *P < 0.05; **P < 0.01; ***P < 0.001; ns = not significant. Data represent three independent experiments and are shown as means (SDs).

To verify the above results, we explored the expression pattern of CD248 in HNSC cell lines, such as CAL27, HSC3, HN6, and SCC-7. qRT-PCR indicated that mRNA expression of CD248 was upregulated in CAL27, HSC3, HN6, and SCC-7 compared to HOK. However, the upregulation in CAL27 and HSC3 was not statistically significant (Figure 10A). Western blot indicated that protein expression of CD248 was higher in CAL27, HN6, and SCC-7 than in HOK (Figures 10B, C). Overall, CD248 expression was upregulated in HNSC cell lines, consistent with the above bioinformatics analysis. In addition, to investigate the effect of CD248 on HNSC cells’ function *in vitro*, two siRNAs (siCD248-1 and siCD248-2) were designed to silence CD248 expression in HN6 cells. The silencing efficiency of siCD248 was verified via Western blotting. CD248 protein expression was significantly lower in both the siCD248-1 and siCD248-2 groups than in the control group (Figures 10D, E), confirming the efficacy of siCD248 silencing. The transwell assay indicated that the invasive cell counts were significantly decreased in both siCD248 groups compared to the control group (Figures 10F, G). The scratch wound healing assay indicated that the migration rate of HN6 cells was significantly repressed by both siCD248 relative to the control group (Figures 10H, I). The EdU assay suggested that siCD248 significantly inhibited the proliferation of HN6 cells compared to the control group (Figures 10J, K). In summary, inhibition of CD248 suppresses the invasion, migration, and proliferation of HN6 cells. In addition, GO enrichment analysis also confirmed that CD248 expression was closely involved in the following biological processes, such as cell proliferation, migration, and adhesion in HNSC (Supplementary Figures S8A). KEGG pathway enrichment analysis indicated that CD248 was related to multiple signaling pathways linked to cell function, including the TGF-β signaling pathway (Hong et al., 2024; Di Benedetto et al., 2018), actin cytoskeleton regulation (Lu S. et al., 2023), and cell adhesion (Kuo et al., 2022; Tomkowicz et al., 2007)in HNSC (Supplementary Figures S8B). Taken together, CD248 plays a crucial role in regulating HNSC functions, including invasive, migratory, and proliferative capabilities, which may facilitate the progression of tumors.

## 4 Discussion

CD248 expression is significantly increased in nine types of cancers in this study. The relationship between upregulated CD248 expression and GBM, KIRC, LIHC, PAAD and STAD has been reported (Lu T. et al., 2023; Gan et al., 2024; Behnan et al., 2017; Simonavicius et al., 2008; Fujii et al., 2015). Conversely, CD248 expression is decreased in ten types of cancers, with the correlation between downregulated CD248 expression and BLCA, PRAD, SKCM, OV, and UCEC having been studied (Kuo et al., 2022; Li et al., 2021; Christian et al., 2008; Matthaiou et al., 2022). The differential expression of CD248 across various tumor types is influenced by multiple factors. First, CD248 expression levels are heterogeneous within distinct tumor microenvironments, with elevated expression observed in tumors rich in CAFs and vascularization, such as in GBM, KIRC, and LIHC (Zhang et al., 2021; Carson-Walter et al., 2009). Teicher et al. indicate that CD248 expression is mainly a perivascular feature in carcinomas (Rouleau et al., 2008). Second, the tissue origin of tumor may also influence CD248 expression, tumors derived from epithelial tissues demonstrate higher CD248 expression, whereas those of mesenchymal origin exhibit relatively lower expression (Rettig et al., 1992). Third, the immune microenvironment also plays a significant role in modulating CD248 expression, with increased CD248 expression detected in immunosuppressive microenvironments, as seen in LIHC, KIRC, DLBC, THYM (Xu et al., 2021; Zhang et al., 2021). Fourth, epigenetic alterations (e.g., DNA methylation) are inversely correlated with the expression level of CD248 (Tserel et al., 2015), Wang et al. indicate that increased CD248 expression might be regulated by hypomethylation of the corresponding enhancer region in breast cancer (Ji et al., 2025). Fifth, tumor subtypes can influence CD248 expression patterns, for example, although CD248 mRNA levels in OV are lower than in normal samples, IHC results in Figure 1H indicate that CD248 is upregulated in endometrioid adenocarcinoma subtype, which accounts for approximately 10%–20% of OV cases. In summary, the differential expression of CD248 across tumors may result from a combination of factors, including the tumor microenvironment, tissue origin, immune microenvironment, epigenetic regulation, and tumor subtypes. Further research into these mechanisms will enhance our understanding of CD248’s role in tumors and promote the development of targeted therapeutic strategies.

CD248 is primarily expressed by stromal cells in the TME, particularly perivascular CAFs and pericytes (Lu et al., 2024). We also confirmed that CD248 expression was positively associated with CAFs infiltration in pan-cancer. In addition, we found that CD248 was also positively associated with infiltration of immune cells, such as macrophages and monocytes, however, CD248 was negatively related to CD4^+^ T cell, CD8^+^ T cell, and Treg infiltration in tumors. Zeng et al. indicate that CD248-expressing CAFs drive recruitment and polarization of macrophages by regulating the expression of C-X-C motif chemokine ligand 12 (CXCL12) (Wu et al., 2022). While Wen et al. demonstrate that CD248-expressing CAFs recruit macrophages by interacting with CD68 and mediate M2 polarization by regulating expression of growth arrest-specific 6 (GAS6) (Yang et al., 2020). In addition, some scholars demonstrate that CD248-expressing CAFs suppress CD8^+^ T cell infiltration by inhibiting CXCL9/10 secretion (Gan et al., 2024). While Lu et al. indicate that CD248-expressing CAFs lined the tumor nest to physically block the infiltration of CD8^+^ T cells (Jiang et al., 2024). Moreover, Franck et al. demonstrate that the expression of CD248 cleaved form does not influence CD8^+^ T cell infiltration, suggesting that CD248 exerts its function primarily through its full-length transmembrane form (Pothin et al., 2025). In summary, the mechanisms by which CD248-expressing CAFs regulate immune cell infiltration are complex and multifactorial, involving intricate interactions within the TME. It has been reported that multiple subsets of CAFs exist, among these, CD248+ mechanoresponsive CAFs represent a specific subset that localizes adjacent to the tumor nest (Jiang et al., 2024). In summary, the overall impact of CAFs on immune cell infiltration is complex and needs to consider both the heterogeneity and interplay among different CAFs subsets within TME.

In addition to immune cells, CD248 expression was positively associated with endothelial cell and hematopoietic stem cell infiltration. Huang et al. indicated that CD248 enhanced tumor angiogenesis by upregulating two proangiogenic factors, OPN and SERPINE1, through the Wnt/β-catenin signaling pathway (Hong et al., 2022); Wu et al. revealed that CD248 promoted melanoma metastasis by regulating cell-fibronectin interaction, migration, and angiogenesis (Kuo et al., 2022); Qin et al. demonstrated that CD248 was specifically expressed in the tumor vasculature and could be used as novel prognostic markers for bladder urothelial carcinoma (Li et al., 2021); Bicknell et al. indicated CD248 on pericytes interacted with CD93 or CLEC14A on endothelial cells via the intermediate multimerin-2 (MMRN-2) in pancreatic cancer (Khan et al., 2017). Because CD248 is widely expressed on perivascular cells, we speculate that CD248 is closely involved in angiogenesis in TME, the mechanism might be that CD248 interferes with stromal cell-extracellular matrix (ECM) interaction or stromal cell-endothelial cell interaction.

Significant efforts have been made to develop novel approaches to targeting CD248 for clinical cancer treatment, including the development of monoclonal antibodies, vaccines, radioimmunotherapy, antibody-drug conjugates (ADCs), and CAR-T. For example, several CD248-specific antibodies have been recently developed for tumor therapy, including monoclonal antibodies such as MORAb-004, hMP-E−8.3, and 3K2L, as well as single-chain variable fragments (scFv) such as scFv78, scFv78-Fc (Li et al., 2014), scFv-CM6, scFv-1C1m, and scFv-7G22. MORAb-004 (also known as ontuxizumab) is the first antibody used in phase I and II clinical trials for treatment-refractory solid tumors such as colorectal carcinoma, sarcoma, mesothelioma, hepatocellular carcinoma, and so on (Diaz et al., 2015; Doi et al., 2019; Norris et al., 2018). However, monoclonal antibodies alone were inadequate to provoke a potent antitumor response in immunosuppressive TME. Even triple therapy comprising MORAb-004 plus gemcitabine/docetaxel (G/D) could not enhance activity over G/D alone in soft-tissue sarcomas (Jones et al., 2019). Hence, other antibody-based combination therapies were invented, such as radioimmunotherapy, vaccines, antibody-drug conjugates (ADCs), and CAR-T.

As for vaccines, Facciabene et al. verified that DNA vaccines targeting CD248 could reduce tumor progression *in vivo* (Facciponte et al., 2014). CD248-specific vaccine plus radiotherapy could further enhance vaccine efficacy compared with either single treatment (Pierini et al., 2021). As for radioimmunotherapy, ^125^I-labeled scFv-1C1m and ^177^Lu labeled scFv-1C1m were used for the treatment of soft-tissue sarcoma (Delage et al., 2021; Delage et al., 2020; D'Onofrio et al., 2021). ^111^In labeled scFv78-Fc was generated to specifically target CD248-positive Ewing sarcoma and neuroblastoma in tumor-bearing mice (Cicone et al., 2020). As for ADCs, antibody-toxin conjugates such as scFv78-Fc-saporin, and hMP-E−8.3-duocarmycin were effective in killing CD248+ sarcoma cells and eradicating sarcoma and osteosarcoma, respectively (Guo et al., 2018; Capone et al., 2017). Multifunctional nanoparticles (NPs)-based drug delivery systems (DDSs) can efficiently and specifically deliver drugs to cancer cells. Hence, numerous studies have sought to develop NP-based DDS and achieve specific delivery of cytotoxic agents into the TME. For example, scFv78-saporin-NPs, namely, antibody-toxin-conjugated NPs, were generated to target CD248-overexpressing solid tumors (Yuan et al., 2017). Additionally, shikonin (SHK)-loaded, scFv78Fc-armed poly (lactic-co-glycolic acid) (PLGA) NPs, namely, scFv78Fc-PLGA-SHK NPs, were also effective for targeted therapy of solid tumors like ovarian cancer (Matthaiou et al., 2022). Beyond that, scFv-CM6-DM1-liposome, an antibody-drug-conjugated immunoliposome, was generated to target CD248-overexpressing human neuroblastoma cells and enhance drug efficacy (Marty et al., 2006). Taken together, NPs-based DDSs might expand the application of the anti-CD248 antibody-based ADCs.

CAR-T therapy is a revolutionary approach to cancer treatment, using genetically modified T cells to precisely target and destroy cancer cells. Modified T cells are engineered for specific antigen recognition, minimizing harm to healthy cells. CAR-T therapy has shown superior effectiveness compared to monoclonal antibodies, offering new hope for patients and highlighting the future of immunotherapy in cancer treatment. To date, anti-CD248 antibodies such as 3K2L, scFv-1C1m, and scFv-7G22 have been applied to construct CAR-T cells for the therapy of breast cancer, lung cancer, and Ewing sarcoma, respectively (Ash et al., 2024; Fierle et al., 2021). Regrettably, there have been no reports on CAR-T therapy using other CD248-specific antibodies, which might also be effective for tumor-targeting therapy. CD248 is scarcely expressed in normal tissues and cells but is significantly upregulated in pericytes and fibroblasts in tumors. These suggest that CD248 is a specific tumor antigen, and targeting CD248 may exhibit on-target/off-tumor activity in pan-cancer. In addition, CD248 is expressed in perivascular fibroblasts of solid tumors, avoiding navigating the harsh immunosuppressive TME. Taken together, CD248 is a promising target for CAR-T therapy in the future. Despite these promising applications, several challenges need to be addressed for successful clinical translation of CD248-targeting therapies. First, potential off-target toxicity may occur due to low but detectable CD248 expression in certain normal tissues. Second, immune-related adverse events, such as cytokine release syndrome (CRS), may arise from CAR-T therapy. Third, tumor heterogeneity can lead to variable treatment responses or adaptive resistance. Fourth, further preclinical and clinical studies are required to evaluate the therapeutic potential of CD248 targeting strategies. To overcome these challenges, it is essential to focus future efforts on optimizing antibody design, exploring combination therapies, and conducting robust preclinical and clinical studies. By addressing these issues, CD248-targeted therapy has the potential to become a cornerstone of future cancer treatment.

Our study reveals that CD248 may function as a candidate biomarker for both prognosis and therapy in pan-cancer. However, there are several limitations in this study. First, our results are largely based on bioinformatics analysis, *in vitro* and *in vivo* experiments are required to confirm our findings and determine the underlying mechanisms. Second, we have only discussed some monoclonal antibodies or combination therapies targeting CD248, whether other more effective CD248-targeting therapies can be translated into clinical treatment for patients remains to be investigated in the future.

## 5 Conclusion

In conclusion, our findings identify CD248 as a potential prognostic and therapeutic biomarker in pan-cancer. CD248 is closely associated with immune cell infiltration and endothelial cell infiltration. CD248 plays a crucial role in HNSC functions, including invasion, migration, and proliferation, while its function in other cancers requires further experimental verification. Immunotherapies targeting CD248, including radioimmunotherapy, vaccines, ADCs, and CAR-T, could be effective treatments for cancer in clinical practice. Moreover, given CD248’s potential role in angiogenesis, the combination of immunotherapy and anti-angiogenic agents might offer a promising approach in cancer treatment in the future.

## Data Availability

The original contributions presented in the study are included in the article/Supplementary Material, further inquiries can be directed to the corresponding author.
