# Supplementary figures and images for: Pan-cancer analysis identified CD248 as a potential target for multiple tumor types
Source: Front Pharmacol. 2025 Apr 10;16:1554632. doi: 10.3389/fphar.2025.1554632 (PMC12018388; doi:10.3389/fphar.2025.1554632)

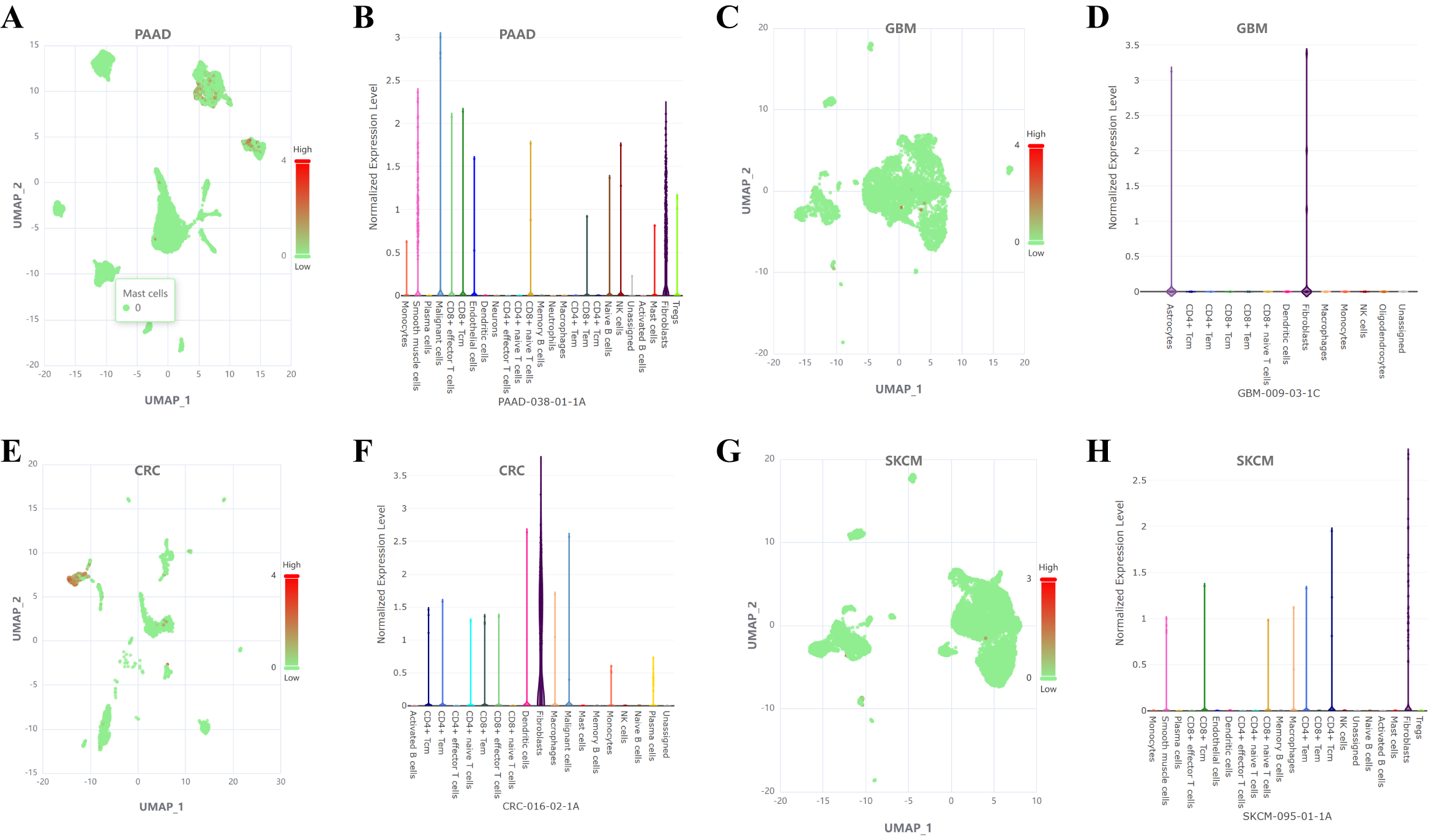

Supplement: Supplementary file 1 [file Image6.tif]

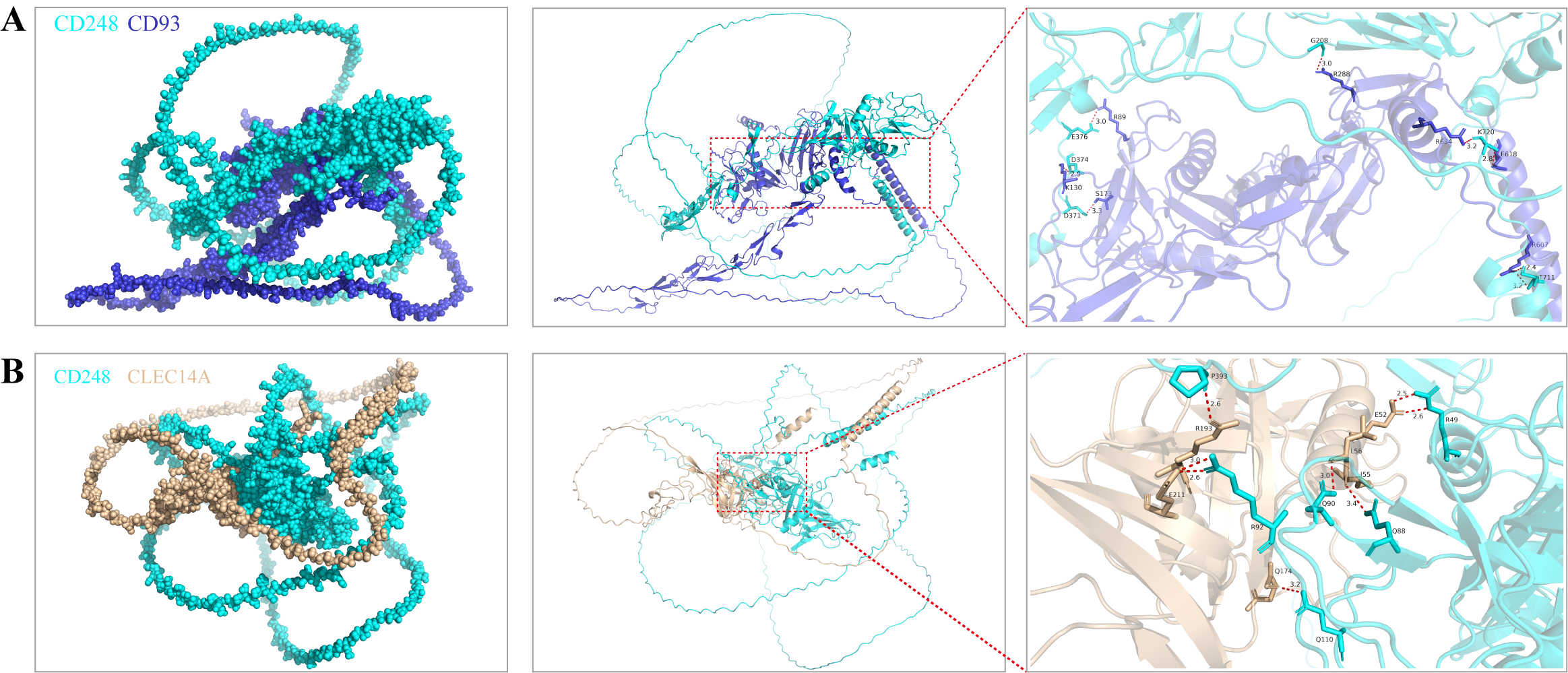

Supplement: Supplementary file 3 [file Image3.tif]

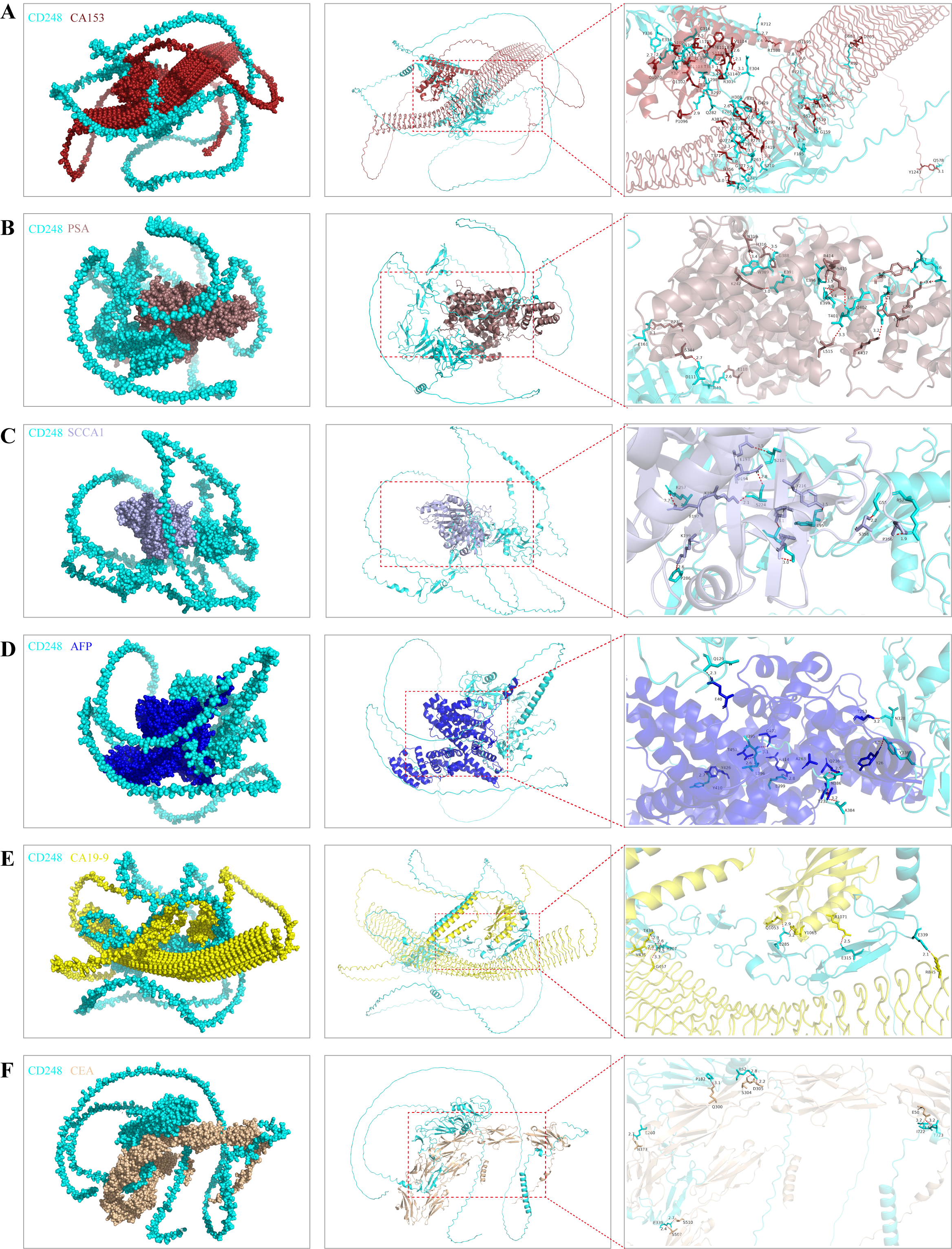

Supplement: Supplementary file 4 [file Image4.tif]

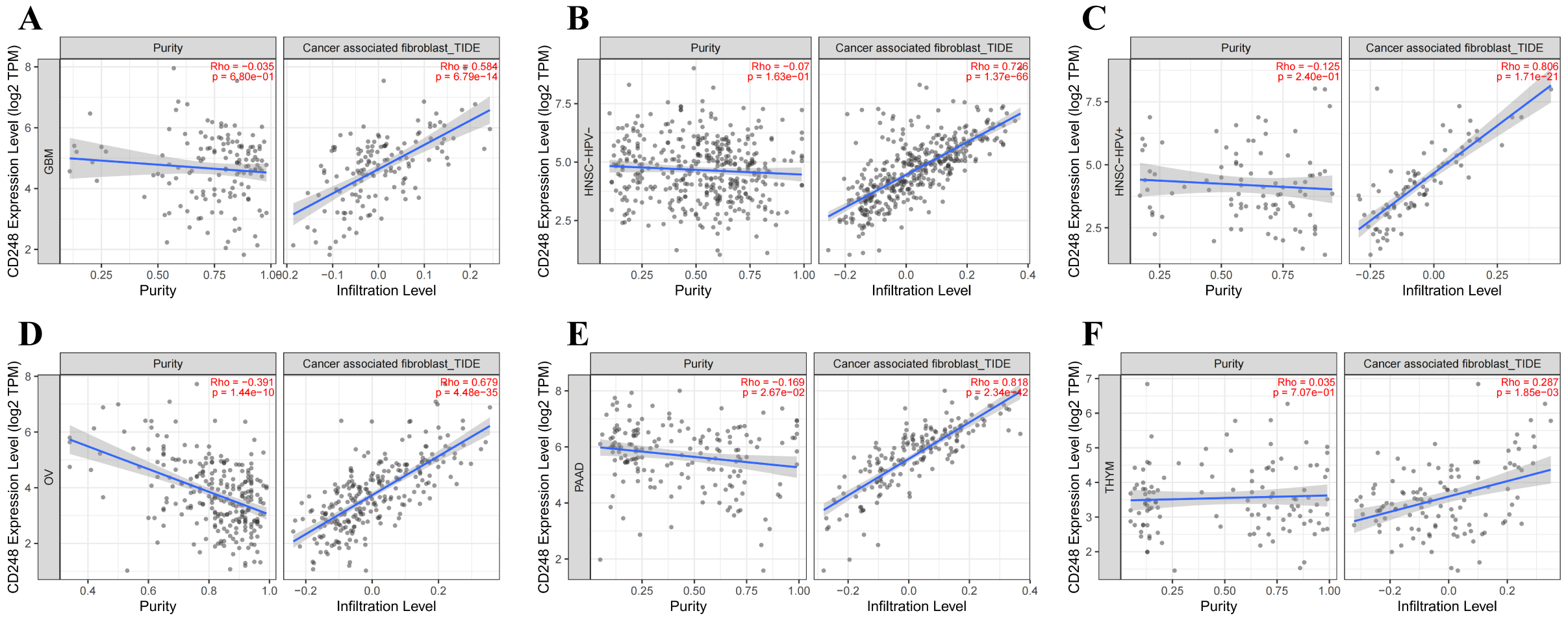

Supplement: Supplementary file 5 [file Image2.tif]

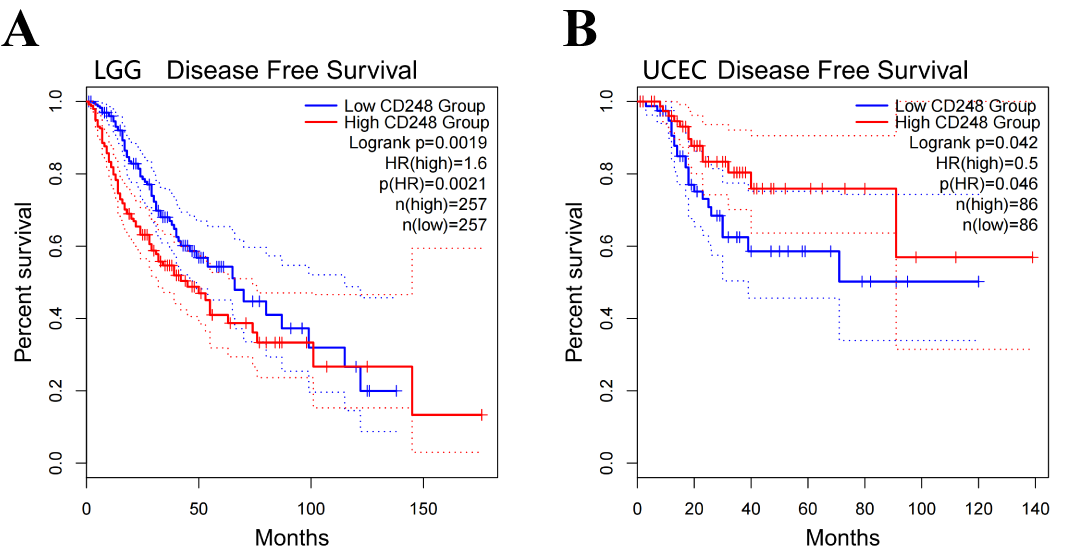

Supplement: Supplementary file 6 [file Image1.tif]

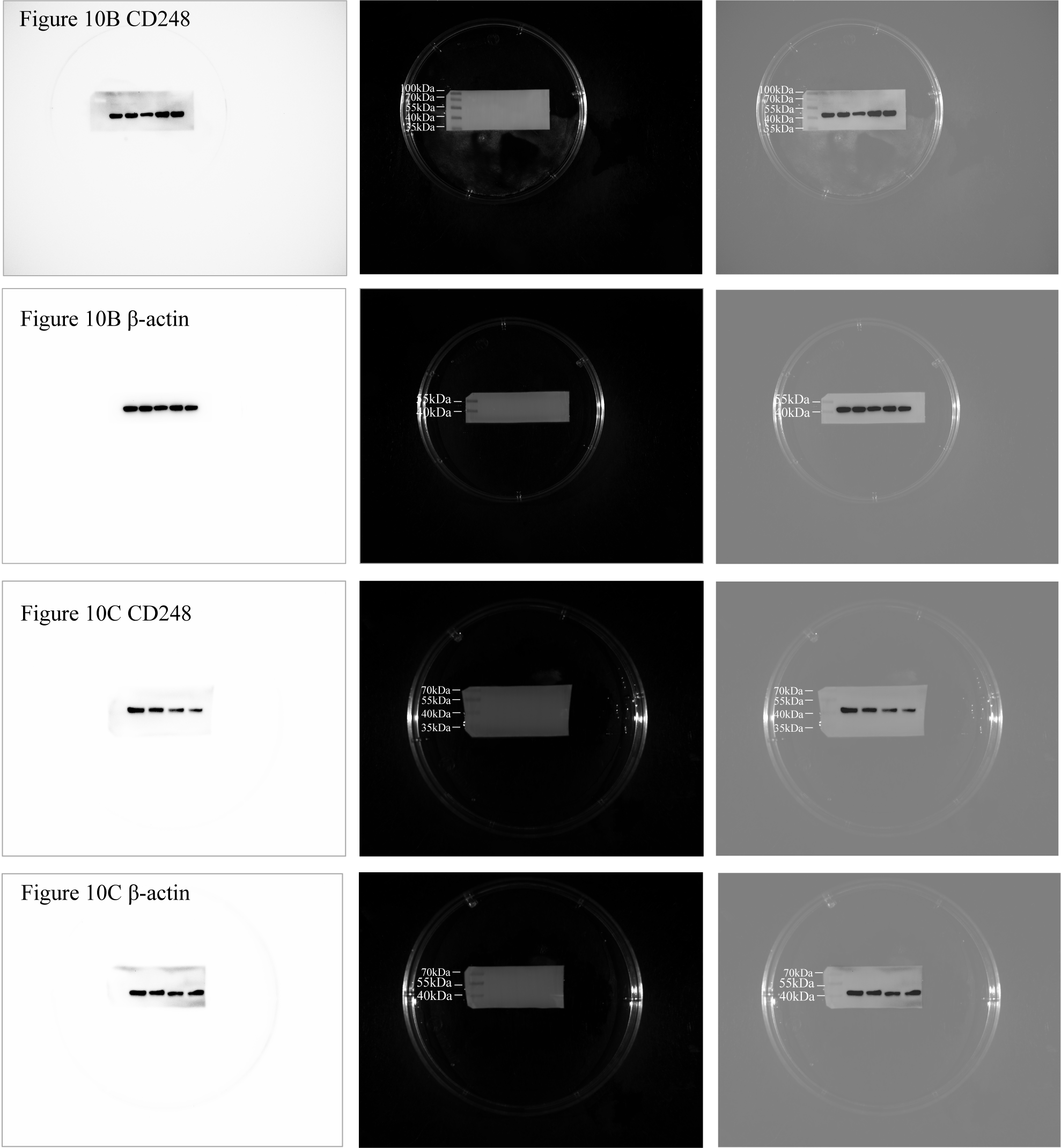

Supplement: Supplementary file 7 [file Image7.tif]

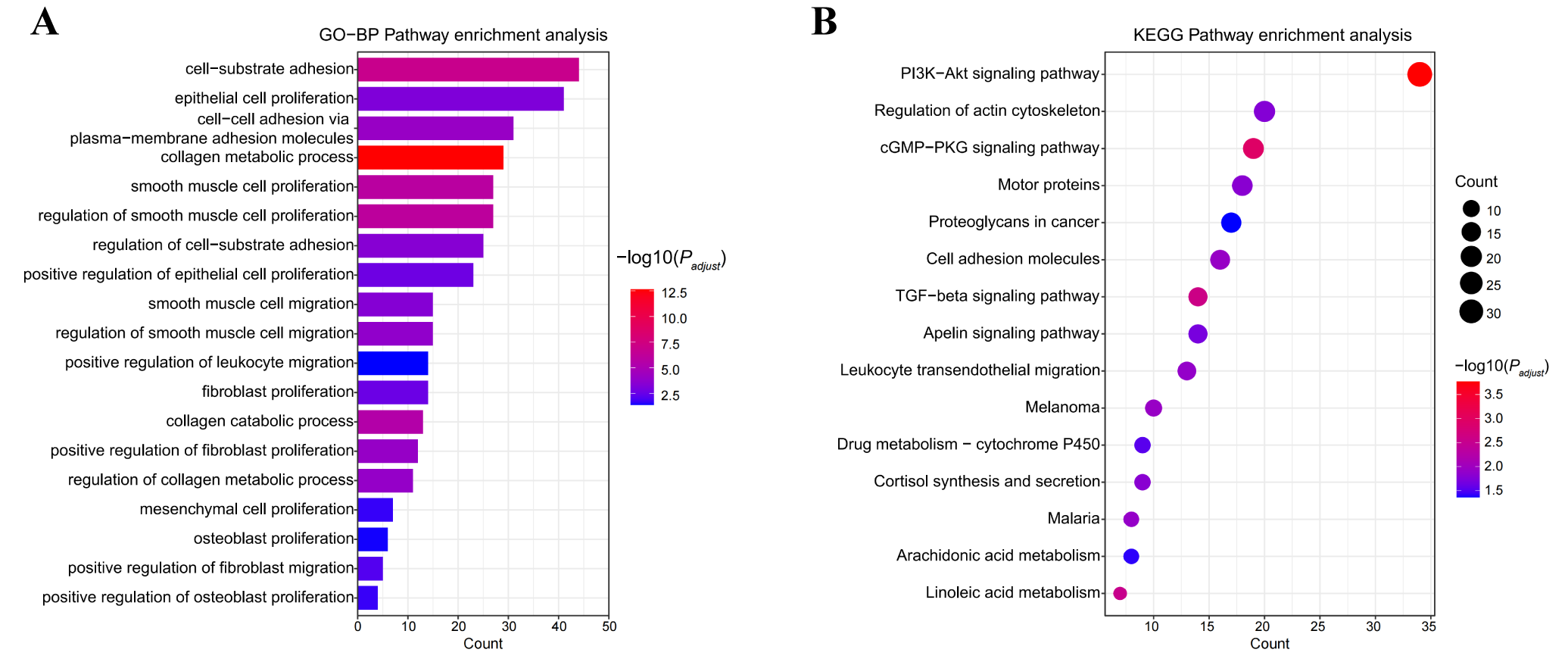

Supplement: Supplementary file 8 [file Image8.tif]

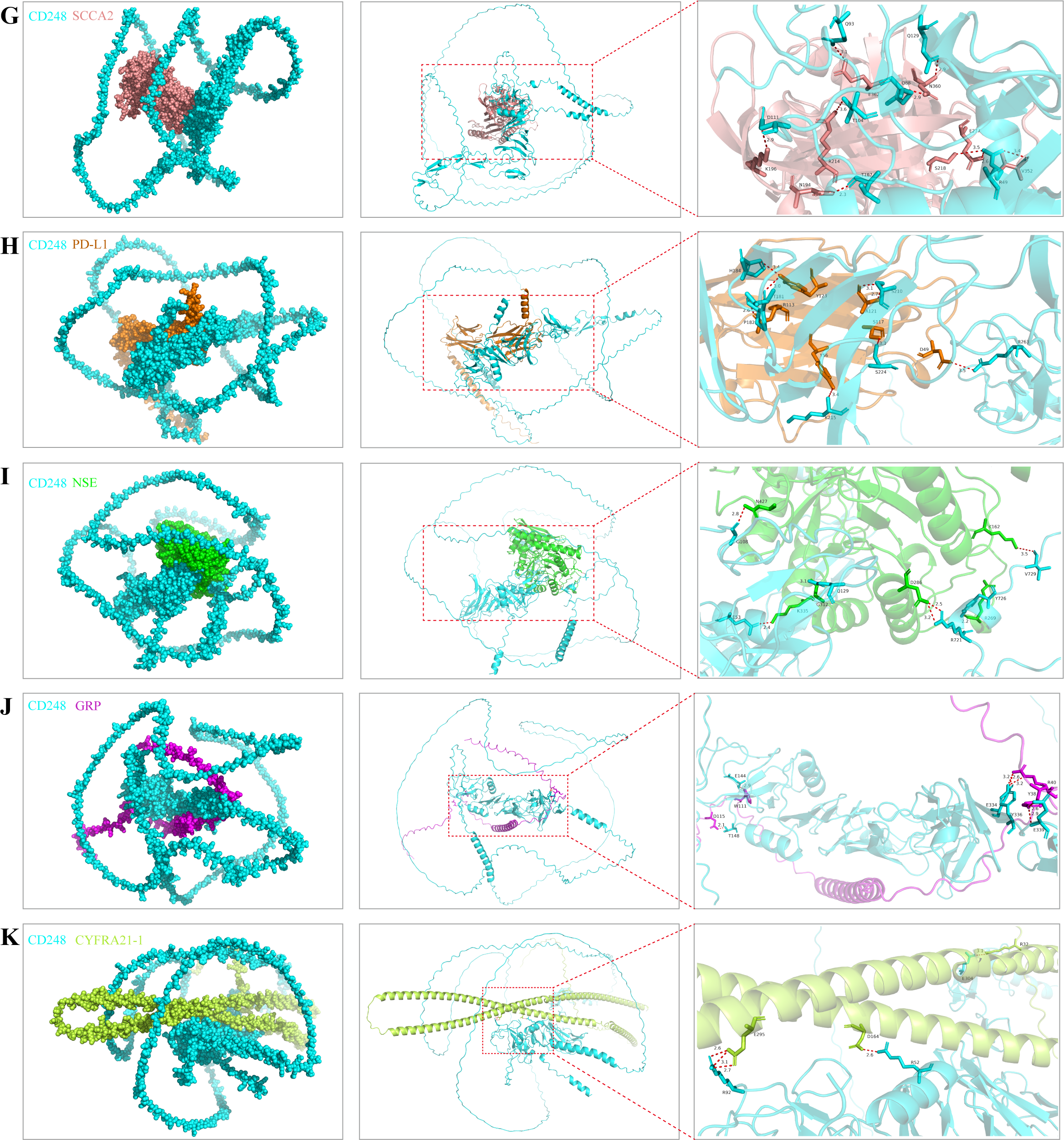

Supplement: Supplementary file 9 [file Image5.tif]
