# Supplementary material for: Pan-cancer analysis identified CD248 as a potential target for multiple tumor types
Source: Front Pharmacol. 2025 Apr 10;16:1554632. doi: 10.3389/fphar.2025.1554632 (PMC12018388; doi:10.3389/fphar.2025.1554632)
Supplement: Supplementary file 2 [file Table1.docx]

**Supplemental materials**

All supplemental materials include Table S1-S5.

**Table S1.** siRNA sequences of human CD248.

| siRNA | Sense (5'-3') | Antisense (5'-3') |
| --- | --- | --- |
| siCD248-1 | CUAUCGCUGGGUCAUCCAU | AUGGAUGACCCAGCGAUAG |
| siCD248-2 | AGAGAGAAUACCGGGUUGG | CCAACCCGGUAUUCUCUCU |

**Table S2.** primer sequences for qRT-PCR

| Gene | Forward primer (5'-3') | Reverse primer (5'-3') | |
| --- | --- | --- | --- |
| CD248 | GCAAGTGGCGAGCACCGCTGGCT | | GGCAGGCGCCCTCGAAGCCA |
| β-actin | AGAAAATCTGGCACCACACCT | | GATAGCACAGCCTGGATAGCA |

**Table S3**. Protein-protein interaction (PPI) network of 50 proteins interacting with CD248

| **Protein** | **Full name of protein** | **Score** |
| --- | --- | --- |
| COL1A1 | Collagen alpha-1(I) chain | 0.525 |
| COL1A2 | Collagen alpha-2(I) chain | 0.509 |
| FBLN2 | Fibulin-2 | 0.469 |
| COL3A1 | Collagen alpha-1(III) chain | 0.453 |
| COL6A2 | Collagen alpha-2(VI) chain | 0.452 |
| PDGFRB | Platelet-derived growth factor receptor beta | 0.419 |
| PCOLCE | Procollagen C-endopeptidase enhancer 1 | 0.358 |
| COL5A1 | Collagen alpha-1(V) chain | 0.357 |
| AEBP1 | Adipocyte enhancer-binding protein 1 | 0.355 |
| MXRA8 | Matrix remodeling-associated protein 8 | 0.348 |
| EMILIN1 | elastin microfibril interfacer 1 | 0.345 |
| COL6A1 | Collagen alpha-1(VI) chain | 0.342 |
| ISLR | Immunoglobulin superfamily containing leucine rich repeat | 0.300 |
| EFEMP2 | EGF containing fibulin extracellular matrix protein 2 | 0.299 |
| COL6A3 | Collagen alpha-3(VI) chain | 0.296 |
| COL5A2 | Collagen alpha-2(V) chain | 0.293 |
| LUM | Lumican | 0.290 |
| TMEM204 | Transmembrane protein 204 | 0.287 |
| BGN | Biglycan | 0.283 |
| FBN1 | Fibrillin-1 | 0.282 |
| RCN3 | Reticulocalbin-3 | 0.282 |
| MFAP4 | Microfibril-associated glycoprotein 4 | 0.278 |
| POSTN | Periostin | 0.276 |
| FMOD | Fibromodulin | 0.266 |
| DCN | Decorin | 0.262 |
| ANGPTL2 | Angiopoietin-related protein 2 | 0.258 |
| CYYR1 | Cysteine and tyrosine rich 1 | 0.258 |
| PRRX1 | Paired mesoderm homeobox protein 1 | 0.257 |
| PDGFRA | Platelet-derived growth factor receptor alpha | 0.254 |
| FBLN5 | Fibulin-5 | 0.250 |
| MRGPRF | Mas-related G-protein coupled receptor member F | 0.250 |
| PLAC9 | Placenta-specific protein 9 | 0.247 |
| SERPINH1 | Serpin H1 | 0.246 |
| CCDC80 | Coiled-coil domain-containing protein 80 | 0.244 |
| FBLN1 | Fibulin-1 | 0.242 |
| MMP2 | matrixmetalloproteinase-2 | 0.236 |
| ADAMTS2 | ADAM metallopeptidase with thrombospondin type 1 motif 2 | 0.235 |
| SLIT3 | Slit homolog 3 protein | 0.235 |
| FKBP10 | FKBP prolyl isomerase 10 | 0.232 |
| LOXL1 | Lysyl oxidase homolog 1 | 0.228 |
| MRC2 | C-type mannose receptor 2 | 0.227 |
| MFAP5 | Microfibrillar-associated protein 5 | 0.226 |
| COL5A3 | Collagen alpha-3(V) chain | 0.221 |
| HSPB6 | Heat shock protein beta-6 | 0.221 |
| THY1 | Thy-1 membrane glycoprotein | 0.221 |
| FSTL1 | Follistatin-related protein 1 | 0.219 |
| ADGRA2 | Adhesion G protein-coupled receptor A2 | 0.218 |
| PODN | Podocan | 0.217 |
| SPARC | SPARC | 0.217 |
| PCDH18 | Protocadherin-18 | 0.216 |

Interaction scores are categorized into confidence levels: low confidence (0.15), medium confidence (0.4), high confidence (0.7), highest confidence (0.9). All data are from STRING tool.

**Table S4**. The correlation between CD248 and tumor mutation burden (TMB)

| **Cancer** | **Correlation** | **Correlation (P)** | |
| --- | --- | --- | --- |
| KIRP | -0.1984 | 0.0009 |  |
| CHOL | -0.4894 | 0.0028 |  |
| LGG | 0.1240 | 0.0044 |  |
| ACC | 0.2236 | 0.0506 |  |
| HNSC | -0.0757 | 0.0903 |  |
| UCEC | -0.0720 | 0.1033 |  |
| LAML | 0.1735 | 0.1850 |  |
| MESO | -0.1511 | 0.1868 |  |
| COAD | 0.0618 | 0.1964 |  |
| ESCA | -0.0950 | 0.1996 |  |
| THCA | -0.0574 | 0.2030 |  |
| UCS | -0.1517 | 0.2599 |  |
| OV | 0.0656 | 0.2743 |  |
| STAD | -0.0543 | 0.2785 |  |
| LIHC | -0.0539 | 0.3043 |  |
| CESC | -0.0575 | 0.3330 |  |
| UVM | -0.1066 | 0.3466 |  |
| SKCM | -0.0378 | 0.4147 |  |
| KICH | 0.0999 | 0.4247 |  |
| TGCT | -0.0525 | 0.5292 |  |
| KIRC | -0.0322 | 0.5351 |  |
| PRAD | -0.0266 | 0.5549 |  |
| DLBC | 0.0847 | 0.5714 |  |
| LUSC | 0.0247 | 0.5886 |  |
| PCPG | -0.0401 | 0.5903 |  |
| BRCA | -0.0158 | 0.6203 |  |
| THYM | -0.0409 | 0.6616 |  |
| GBM | -0.0319 | 0.6901 |  |
| BLCA | -0.0185 | 0.7112 |  |
| READ | 0.0148 | 0.8560 |  |
| LUAD | -0.0065 | 0.8822 |  |
| SARC | 0.0047 | 0.9426 |  |
| PAAD | 0.0000 | 0.9997 |  |

Notes: All abbreviations and their corresponding full names of cancers are shown in Table 1.

**Table S5.** The correlation between CD248 and microsatellite instability (MSI)

| **Cancer** | **Correlation** | **Correlation (P)** |
| --- | --- | --- |
| KIRC | -0.1689 | 0.0008 |
| TGCT | 0.2147 | 0.0073 |
| READ | -0.1944 | 0.0168 |
| KIRP | -0.1297 | 0.0215 |
| BLCA | -0.0826 | 0.0883 |
| STAD | -0.0797 | 0.0948 |
| BRCA | -0.0456 | 0.1210 |
| HNSC | -0.0661 | 0.1246 |
| PRAD | -0.0609 | 0.1535 |
| LIHC | -0.0690 | 0.1595 |
| PAAD | -0.0969 | 0.1958 |
| UCS | -0.1438 | 0.2858 |
| ESCA | -0.0748 | 0.3002 |
| DLBC | 0.1255 | 0.3953 |
| CESC | 0.0484 | 0.4042 |
| THCA | 0.0273 | 0.5235 |
| THYM | -0.0552 | 0.5480 |
| MESO | 0.0645 | 0.5622 |
| LUSC | -0.0226 | 0.6055 |
| SKCM | 0.0534 | 0.6334 |
| KICH | 0.0479 | 0.6560 |
| UCEC | 0.0182 | 0.6698 |
| SARC | 0.0205 | 0.7447 |
| LGG | -0.0137 | 0.7538 |
| UVM | 0.0317 | 0.7800 |
| OV | 0.0135 | 0.8125 |
| COAD | 0.0107 | 0.8193 |
| ACC | 0.0256 | 0.8237 |
| GBM | -0.0171 | 0.8265 |
| LUAD | -0.0090 | 0.8297 |
| PCPG | -0.0055 | 0.9411 |
| CHOL | 0.0069 | 0.9648 |

Note: All abbreviations and their corresponding full names of cancers are shown in Table 1.
